# A computationally efficient algorithm for genomic prediction using a Bayesian model

**DOI:** 10.1186/s12711-014-0082-4

**Published:** 2015-04-30

**Authors:** Tingting Wang, Yi-Ping Phoebe Chen, Michael E Goddard, Theo HE Meuwissen, Kathryn E Kemper, Ben J Hayes

**Affiliations:** Faculty of Science, Technology and Engineering, La Trobe University, Melbourne, VIC 3086 Australia; Biosciences Research Division, Department of Primary Industries, Bundoora, Melbourne, VIC 3083 Australia; Dairy Futures Cooperative Research Centre, Bundoora, Melbourne, VIC 3083 Australia; Faculty of Veterinary and Agricultural Sciences, University of Melbourne, Parkville, Melbourne, VIC 3052 Australia; Institute Animal and Aquacultural Sciences, Norwegian University of Life Science, Box 5003, As, N1432 Norway

## Abstract

**Background:**

Genomic prediction of breeding values from dense single nucleotide polymorphisms (SNP) genotypes is used for livestock and crop breeding, and can also be used to predict disease risk in humans. For some traits, the most accurate genomic predictions are achieved with non-linear estimates of SNP effects from Bayesian methods that treat SNP effects as random effects from a heavy tailed prior distribution. These Bayesian methods are usually implemented via Markov chain Monte Carlo (MCMC) schemes to sample from the posterior distribution of SNP effects, which is computationally expensive. Our aim was to develop an efficient expectation–maximisation algorithm (emBayesR) that gives similar estimates of SNP effects and accuracies of genomic prediction than the MCMC implementation of BayesR (a Bayesian method for genomic prediction), but with greatly reduced computation time.

**Methods:**

emBayesR is an approximate EM algorithm that retains the BayesR model assumption with SNP effects sampled from a mixture of normal distributions with increasing variance. emBayesR differs from other proposed non-MCMC implementations of Bayesian methods for genomic prediction in that it estimates the effect of each SNP while allowing for the error associated with estimation of all other SNP effects. emBayesR was compared to BayesR using simulated data, and real dairy cattle data with 632 003 SNPs genotyped, to determine if the MCMC and the expectation-maximisation approaches give similar accuracies of genomic prediction.

**Results:**

We were able to demonstrate that allowing for the error associated with estimation of other SNP effects when estimating the effect of each SNP in emBayesR improved the accuracy of genomic prediction over emBayesR without including this error correction, with both simulated and real data. When averaged over nine dairy traits, the accuracy of genomic prediction with emBayesR was only 0.5% lower than that from BayesR. However, emBayesR reduced computing time up to 8-fold compared to BayesR.

**Conclusions:**

The emBayesR algorithm described here achieved similar accuracies of genomic prediction to BayesR for a range of simulated and real 630 K dairy SNP data. emBayesR needs less computing time than BayesR, which will allow it to be applied to larger datasets.

**Electronic supplementary material:**

The online version of this article (doi:10.1186/s12711-014-0082-4) contains supplementary material, which is available to authorized users.

## Background

Genomic prediction uses information from high-density genetic polymorphisms, such as single nucleotide polymorphisms (SNP) panels, to predict the genetic merit of individuals for quantitative traits. Selection based on these estimated breeding values could substantially increase the rates of genetic improvement for quantitative traits in animal and plant species [1]. Implementation of genomic selection is a two-step process: (1) estimation of the effects of SNPs in a reference population given the phenotypes and SNP genotypes of reference individuals and (2) calculation of genomic estimated breeding values (GEBV) for selection candidates based on their genotypes [1]. If the SNP effects are random variables drawn from a prior distribution, the accuracy of GEBV is maximised if, in step (1), SNP effects are estimated by their expected value conditional on the data.

Several methods, which differ in the assumed prior distribution of SNP effects, have been proposed to estimate SNP effects for genomic prediction. The prior assumption that SNP effects are all drawn from the same normal distribution results in the statistical method called best linear unbiased prediction (BLUP). BLUP for genomic prediction can be implemented using two equivalent models [2]. Either the SNP effects are estimated directly, termed SNP_BLUP (e.g. [1]), or a genomic relationship matrix is calculated from SNP genotypes, termed genomic BLUP (GBLUP) [2,3]. Other models assume that the SNP effects follow a non-normal distribution. For example, in the model called BayesA, the SNP effects follow a Student’s t distribution [1], while mixture distributions are used in BayesB [1], BayesC, BayesCπ [4] and BayesR [5], and exponential distributions are used in BayesLASSO [6]. With real data and for some traits, GBLUP methods achieve levels of accuracy of genomic prediction similar to non-normal distributions methods such as BayesA, BayesB, and BayesR when moderate SNP densities (e.g. 50 K in dairy cattle; less in some crop species with extensive linkage disequilibrium) were used [7-11]. As described by several authors, GBLUP has the advantage that it is computationally efficient [12-14]. However, for traits with quantitative traits loci (QTL) of large to moderate effect, the Bayesian methods can give higher accuracies of prediction than GBLUP [15-17]. Moreover, genomic prediction models that assume non-normal distributions of effects in some cases give higher accuracies than GBLUP when very large numbers of SNPs (e.g. 630 K or whole-genome sequence data) are used, particularly for multi-breed and across-breed predictions [5,18-22]. A disadvantage of these methods, however, is that it is difficult, if not impossible, to write closed form solutions for estimates of SNP effects or other parameters, so Markov chain Monte Carlo (MCMC) sampling is used to derive posterior distributions for these effects (e.g. [1]). However, this is computationally expensive, particularly when the number of SNPs is large. For example, the BayesB method can result in the highest accuracy of genomic prediction in some situations, but, since it uses a Metropolis Hastings algorithm, computing time with large numbers of SNPs (e.g. 800 000 SNPs) is very long. Other methods, such as BayesA, BayesLASSO, and BayesR, are usually implemented using Gibbs sampling. While Gibbs sampling is faster than the Metropolis Hasting algorithm, it is still slow with very large numbers of SNPs genotyped in large numbers of individuals.

In dairy cattle routine genomic evaluations, different genomic prediction methods have been implemented by different countries and organisations [23]. According to Mantysaari [23], GBLUP, or its single-step implementation [24,25], is one of the most popular genomic prediction methods implemented for official genomic evaluation in many countries, including Canada, New Zealand, Australia, Germany and Ireland. By contrast, only two countries, i.e. The Netherlands and Switzerland have implemented MCMC non-linear models (BayesA and BayesC) for genomic prediction. In addition, non MCMC versions of BayesA (also termed nonlinear A [2]) are used for genomic prediction in the USA. In the future, genomic evaluations may be based on whole-genome sequence data and Bayesian methods may be required to take advantage of this data [26,27]. Therefore, a way to implement Bayesian models that is faster to compute than the MCMC methods is desirable.

There have been a number of proposals to reduce the computing time required to arrive at satisfactory estimates of the SNP effects from Bayesian methods (e.g. [28-30]). These proposals use algorithms other than Gibbs sampling. For instance, VanRaden [2] described an iterative method to implement approximations of both BayesA and BayesB. Meuwissen [29] described a method termed fastBayesB by using iterative conditional expectation (ICE) in the BayesLASSO model. FastBayesB iteratively calculated each SNP’s posterior mean, conditioning on current estimates of all other SNPs as if they were true effects. FastBayesB greatly reduces computing time but several parameters required to describe the prior distribution of SNP effects are assumed to be known. This issue was dealt with in a later publication by an expectation-maximisation (EM) algorithm that estimated those parameters by maximising a joint posterior probability based on the prior distribution of SNP effects, in a method called EmBayesB [31]. Lower prediction accuracies were observed for these methods compared with MCMC implementations [29,31]. Two potential reasons for this are: (1) the errors in the estimates of SNP effects other than the SNP for which the effect is being estimated were ignored [29], and (2) the prior distribution of SNP effects that they assume (a double exponential) may not match the true distribution of SNP effects as well as the mixture distribution assumed by BayesB and BayesR.

Our aim in this paper was to develop a fast EM counterpart to MCMC BayesR (emBayesR). BayesR assumes that SNP effects are drawn from a mixture of normal distributions, one with zero variance (and hence zero effects). BayesR shares some of the advantages of BayesB, in that SNP effects can be zero, moderate, or large, but is more computationally efficient since it can be implemented with Gibbs sampling [5]. In BayesR, the proportion of SNPs in each normal distribution is estimated from the data, instead of being pre-set as a constant value in BayesB. Consequently, BayesR is able to approximate a wide range of possible true distributions of SNP effects. With real data, BayesR achieves accuracies comparable to BayesA [5] and BayesB (Goddard and Meuwissen, unpublished data).

Our EM algorithm retains the BayesR model assumption that SNP effects are assumed to be derived from four different normal distributions, but requires much less computing time than BayesR. It also differs from other EM methods by estimating the effect of each SNP while accounting for the errors in the estimates of all other SNPs. It does this by treating the combined effect of the other SNPs as a residual breeding value, and approximating its prediction error variance from a GBLUP prediction. To compare speed and accuracy of prediction of emBayesR with that from BayesR, we used both a simulated dataset and a real dataset on 630 K SNPs for dairy cattle.

## Methods

In this section, we first describe the model of BayesR (here also named MCMC_BayesR) for genomic prediction and second, an EM algorithm named emBayesR. Finally, the 10 K simulated data and 630 K real dairy data that were used to evaluate the performance of emBayesR, are described.

### Statistical model for emBayesR and prior distributions of parameters

The linear model for phenotypes is:

1 $$ \mathbf{y}={1}_{\boldsymbol{n}}\mu + \mathbf{Zg}+\mathbf{e}, $$

where, **y** is a *n* × 1 vector of phenotypic records (*n* is the number of animals); **1**_***n***_ is a *n* x *1* vector of 1 s, *μ* is the population mean; **Z** is a *n* × *m* design matrix with elements ${\mathbf{Z}}_{\mathrm{i}}=\left({\mathbf{x}}_{\mathrm{i}}-2{p}_i\right)/\sqrt[2]{2{p}_i\left(1-{p}_i\right)}$, in which **x**_i_ is the *n* × 1 vector of genotypes for the *i*^*th*^ SNP (0, 1 or 2 copies of the second allele), and *p*_*i*_ is the allele frequency of each SNP *i* (m is the number of SNPs); **e** is a *n* × 1vector of random normal deviates, $\mathbf{e}\sim N\left(0,\mathbf{I}{\sigma}_e^2\right)$; **g** is a *m* × 1 vector of SNP effects.

For convenience, polygenic effects were not included in the model but they can be readily added (and have been added in the MCMC version of BayesR, e.g. [5]).

BayesR [5] assumes that SNP effects (**g**) are drawn from a mixture of four normal distributions $\mathrm{N}\left(0,\ {\boldsymbol{\upsigma}}_k^2\right)$ according to the proportion vector **Pr** = {*Pr*_*k*_|*k* = 1, 2, 3, 4}. Variances used were ${\boldsymbol{\upsigma}}_{\mathbf{k}}^2=\left\{0,\ 0.0001\kern0.5em *\kern0.5em {\sigma}_g^2,0.001\kern0.5em *\kern0.5em {\sigma}_g^2,0.01\kern0.5em *\kern0.5em {\sigma}_g^2\right\}$ for the analysis of the real dairy data and ${\boldsymbol{\upsigma}}_{\mathbf{k}}^2=\left\{0,\ 0.0006\kern0.5em *\kern0.5em {\sigma}_g^2,0.006\kern0.5em *\kern0.5em {\sigma}_g^2,0.06\kern0.5em *\kern0.5em {\sigma}_g^2\right\}$ for the analysis of the simulated data, where ${\sigma}_g^2$ is total genetic variance [5]. Here, the coefficients of ${\sigma}_g^2$ used to define ${\boldsymbol{\upsigma}}_{\mathbf{k}}^2$ for the simulated data were different to those used for the real data because of the criterion that the sum of the variance across all SNPs approaches the overall genetic variance explained by SNPs. In the simulation data, with 10 050 SNPs, there were only 50 QTL (17 QTL in ${\sigma}_k^2\left[2\right]$, 16 QTL in ${\sigma}_k^2\left[3\right]$ and 17 QTL in ${\sigma}_k^2\left[4\right]$). To make the overall variance summed over all the SNPs approximately equal to ${\sigma}_g^2$, vector ${\boldsymbol{\upsigma}}_{\mathbf{k}}^2$ for the simulated data was set to $\left\{0,\ 0.0006\kern0.5em *\kern0.5em {\sigma}_g^2,0.006\kern0.5em *\kern0.5em {\sigma}_g^2,0.06\kern0.5em *\kern0.5em {\sigma}_g^2\right\}$. For the real data (with high-density SNP panels), the value of ${\boldsymbol{\upsigma}}_{\mathbf{k}}^2$ that is $\left\{0,\ 0.0001\kern0.5em *\kern0.5em {\sigma}_g^2,0.001\kern0.5em *\kern0.5em {\sigma}_g^2,0.01\kern0.5em *\kern0.5em {\sigma}_g^2\right\}$ was assumed as in [5]. In addition, the proportion of SNPs in each normal distribution $\left(P{r}_k;\kern0.5em {\varSigma}_{k\kern0.5em =\kern0.5em 1}^4\kern0.5em P{r}_k=1\right)$ was assumed to follow a Dirichlet distribution with parameter **α** = (1, 1, 1, 1)^*T*^, which is a 4 × 1 vector of the pseudo-counts of the number of SNPs in each distribution. Therefore, the BayesR model has two fixed parameters as input: ${\boldsymbol{\upsigma}}_{\mathbf{k}}^2$ and **α** (the prior for **Pr**).

For each SNP *i*, there is a latent binary variable *b*_*ik*_ (*b*_*ik*_ = 0 or 1) that indicates whether or not the effect of SNP *i* follows the normal distribution with variance ${\boldsymbol{\upsigma}}_{\boldsymbol{k}}^2\kern0.5em \left(k = 1,\ 2, 3, 4\right)$. Therefore:

2 $$ p\left({b}_{ik}=1\Big|P{r}_k\right)=P{r}_k $$

Then, the prior distribution of each SNP effect (*g*_*i*_) conditional on variable *b*_*ik*_ is:

3 $$ p\left({g}_i\Big|{b}_{ik}\right)=\left\{\begin{array}{c}\hfill \frac{1}{\sqrt{2\pi {\sigma}_k^2}} \exp \left(-\frac{g_i^2}{2{\sigma}_k^2}\right),\kern4.25em \mathrm{if}\ {b}_{ik}=1\ \left(k=2,3,4\right)\hfill \\ {}\hfill \delta \left({g}_i\right),\kern10em \mathrm{if}\kern0.5em {b}_{i1}=1\hfill \end{array}\right.\kern5em , $$

where *δ*(*g*_*i*_) denotes the Dirac delta function with all probability mass at *g*_*i*_ = 0*.*

Then, the joint distribution *p*(*g*_*i*_, **b**_**i**_) conditional on **Pr** is:

4 $$ \begin{array}{l}p\left({g}_i,{\mathbf{b}}_{\mathbf{i}}\Big|\mathbf{Pr}\right)={\displaystyle \prod_{k=1}^4}p\left({g}_i\Big|{b}_{ik}\right)\times p\left({b}_{ik}\Big|P{r}_k\right)\\ {}=\kern0.5em {\left(\updelta \left({g}_i\right)P{r}_1\right)}^{b_{i1}}\kern0.5em {\prod}_{k=2}^4{\left(\frac{1}{\sqrt{2\uppi {\sigma}_k^2}} \exp \left(-\frac{g_i^2}{2{\sigma}_k^2}\right)P{r}_k\right)}^{b_{ik}}\end{array} $$

### Expectation-maximisation steps for emBayesR

An EM algorithm is applied to BayesR to obtain estimates of parameters, including SNP effects (**ĝ**) and the proportion of SNP effects in each distribution $\left(\widehat{\mathbf{Pr}}\right)$. The aim of emBayesR is to predict **Zg** by **Zĝ** as accurately as possible. The best predictor for *g*_*i*_ would be *g*_*i*_ = E(*g*_*i*_|**y**), but we approximated this by estimating *ĝ*_*i*_ by the value of *g*_*i*_ that maximises the posterior probability $\mathrm{P}\left({g}_i\Big|\mathbf{y},\kern0.5em \widehat{\mathbf{Pr}},\kern0.5em \widehat{\mu},\kern0.5em \widehat{\sigma_e^2}\right)$, where $\widehat{\mathbf{Pr}}$**,**$\widehat{\mu}$ and $\widehat{\sigma_e^2}$ are the MAP (Maximum A Posterior) estimator of **Pr**, *μ*, and ${\sigma}_e^2$, conditional on **y**. In the following, we first deal with estimating *ĝ*_*i*_ and then return to $\widehat{\mathbf{Pr}}$.

For estimation of *g*_*i*_, we maximised the marginal posterior of *g*_*i*_ rather than the joint posterior of all **g**. To do this, we first introduce two vectors of missing data (**u**, **b**_***i***_), and use the EM algorithm to integrate them out of the posterior distributions. Here, **u** is the combined effects of all other SNPs except the current SNP, i.e. **u** = **Zg** − **Z**_***i***_*g*_*i*_, and the other vector **b**_**i**_ = {*b*_*ik*_|*k* = 1, 2, 3, 4} is for indicator variables that determine which normal distribution each SNP effect is derived from, as described above. Then Equation (1) can be re-written as:

5 $$ \mathbf{y}={1}_{\boldsymbol{n}}\mu +{\mathbf{Z}}_{\mathrm{i}}{g}_i+\mathbf{u}+\mathbf{e}. $$

The full posterior distribution with the missing data, $p\left({g}_i,\mathbf{u},\mu, {\mathbf{b}}_{\boldsymbol{i}}\Big|\mathbf{y},\ \widehat{\mathbf{Pr}}\right)$ is (following Bayes’ theorem):

6 $$ \begin{array}{l}p\left({g}_i,\mathbf{u},{\mathbf{b}}_{\boldsymbol{i}}\Big|\mathbf{y},\kern0.5em \widehat{\mu},\kern0.75em \widehat{\sigma_e^2},\ \widehat{\mathbf{Pr}}\right) = \frac{f\left(\mathbf{y}\Big|{g}_i,\mathbf{u},\widehat{\mu},\kern0.75em \widehat{\sigma_e^2},\ \widehat{\mathbf{Pr}}\right)p\left({g}_i,{\mathbf{b}}_{\boldsymbol{i}}\Big|\widehat{\mathbf{Pr}}\right)}{p\left(\mathbf{y},\mathbf{u}\right)}\\ {}\propto f\left(\mathbf{y}\Big|{g}_i,\mathbf{u},\widehat{\mu},\kern0.75em \widehat{\sigma_e^2},\ \widehat{\mathbf{Pr}}\right)p\left({g}_i,{\mathbf{b}}_{\boldsymbol{i}}\Big|\widehat{\mathbf{Pr}}\right)\end{array} $$

Where

$$ f\left(\mathbf{y}\Big|{g}_i,\mathbf{u},\widehat{\mu},\kern0.75em \widehat{\sigma_e^2},\ \widehat{\mathbf{Pr}}\right)=\frac{1}{{\left(2\pi \kern0.5em \widehat{\sigma_e^2}\right)}^{\frac{n}{2}}} exp\left[-\frac{1}{\widehat{\sigma_e^2}}{\left({\mathbf{y}}^{*}-\mathbf{u}-{\mathbf{Z}}_{\mathrm{i}}{g}_i\right)}^{\hbox{'}}\left({\mathbf{y}}^{*}-\mathbf{u}-{\mathbf{Z}}_{\mathrm{i}}{g}_i\right)\right] $$

is the likelihood of the data given y^*^ and **u**, and ${\mathbf{y}}^{*}=\mathbf{y}-{1}_{\boldsymbol{n}}\widehat{\mu}$. Then, the log of the posterior is:

$$ logp\left({g}_i,\mathbf{u},{\mathbf{b}}_{\boldsymbol{i}}\Big|\mathbf{y},\widehat{\mu},\kern0.75em \widehat{\sigma_e^2},\ \widehat{\mathbf{Pr}}\right)= logf\left(\mathbf{y}\Big|{g}_i,\mathbf{u},\widehat{\mu},\kern0.75em \widehat{\sigma_e^2},\ \widehat{\mathbf{Pr}}\right)+ logp\left({g}_i,{\mathbf{b}}_{\boldsymbol{i}}\Big|\ \widehat{\mathbf{Pr}}\right)+ constant $$

This can be re-written as:

6a $$ logf\left(\mathbf{y}\Big|{g}_i,\mathbf{u},\widehat{\mu},\kern0.75em \widehat{\sigma_e^2},\ \widehat{\mathbf{Pr}}\right)=-0.5 nlog\widehat{\sigma_e^2}-\frac{1}{2\widehat{\sigma_e^2}}{\left({\mathbf{y}}^{*}-\mathbf{u}-{\mathbf{Z}}_{\mathrm{i}}{g}_i\right)}^{\hbox{'}}\left({\mathbf{y}}^{*}-\mathbf{u}-{\mathbf{Z}}_{\mathrm{i}}{g}_i\right) $$

6b $$ logp\left({g}_i,{\mathbf{b}}_{\boldsymbol{i}}\Big|\widehat{\mathbf{Pr}}\right)={b}_{i1} log\left(\updelta \left({g}_i\right){\widehat{Pr}}_1\right)+{\displaystyle \sum_{k=2}^4}{b}_{ik}\left(-\frac{1}{2} log{\sigma}_k^2-\frac{g_i^2}{2{\sigma}_k^2}+ log{\widehat{Pr}}_k\right). $$

In the E-step of emBayesR, we will take expectation of the log posterior function of Equation (6) over the missing data (**u**, **b**). Only the second term (6b) in the equation $logp\left({\mathrm{g}}_{\mathrm{i}},\mathbf{u},{\mathbf{b}}_{\boldsymbol{i}}\Big|\mathbf{y},\widehat{\mu},\kern0.75em \widehat{\sigma_e^2},\ \widehat{\mathbf{Pr}}\right)$ involves **b**_***i***_. Therefore:

$$ \begin{array}{l}{\mathrm{E}}_{{\mathbf{b}}_{\boldsymbol{i}}} log p\left({g}_{\mathrm{i}},\ {\mathbf{b}}_{\boldsymbol{i}}\Big|\widehat{\mathbf{Pr}}\right)=\\ {}{\mathrm{E}}_{{\mathbf{b}}_{\boldsymbol{i}}}\left[{b}_{i1} log\left(\delta \left({g}_i\right){\widehat{Pr}}_1\right)+{\displaystyle \sum_{k=2}^4}{b}_{ik}\left(-\frac{1}{2} log{\sigma}_k^2-\frac{g_i^2}{2{\sigma}_k^2}+ log{\widehat{Pr}}_k\right)\right]\\ {}={P}_{i1} log\left(\delta \left({g}_i\right){\widehat{Pr}}_1\right)+{\sum}_{k=2}^4{P}_{ik}\left(-\frac{1}{2} log{\sigma}_k^2-\frac{g_i^2}{2{\sigma}_k^2}+ log{\widehat{Pr}}_k\right)\end{array} $$

where ${P}_{ik}=\mathrm{E}\left({b}_{ik}\Big|\mathbf{y},{\widehat{Pr}}_k\right)$, which is the posterior probability for each SNP to belong to each of the four normal distributions. The derivation of *P*_*ik*_ is explained in Additional file 1.

Next, we take the expectation over missing data **u**. Only the quadratic form **Q** = (y* − **u** − **Z**_i_*g*_*i*_) ' (y* − **u** − **Z**_i_*g*_*i*_) in the first term of Equation (6a) is related to **u**. To calculate the expectation of Equation (6a) over **u**, we only need to take the expectation of **Q** over **u**. Applying Searle’s expectation rule [32] to E_û_(**Q**), we obtain:

$$ \begin{array}{l}{\mathrm{E}}_{\widehat{\mathrm{u}}}\left(\mathbf{Q}\right)={\mathrm{E}}_{{}_{\widehat{\mathrm{u}}}}\left[{\left({\mathbf{y}}^{*}-\mathbf{u}-{\mathbf{Z}}_{\mathrm{i}}{g}_i\right)}^{\hbox{'}}\left({\mathbf{y}}^{*}-\mathbf{u}-{\mathbf{Z}}_{\mathrm{i}}{g}_i\right)\right]\\ {}={\left({\mathbf{y}}^{*}-\widehat{\mathbf{u}}-{\mathbf{Z}}_{\mathrm{i}}{g}_i\right)}^{\hbox{'}}\left({\mathbf{y}}^{*}-\widehat{\mathbf{u}}-{\mathbf{Z}}_{\mathrm{i}}{g}_i\right)+tr\left(\mathrm{P}\mathrm{E}\mathrm{V}\left(\widehat{\mathbf{u}}\right)\right), \end{array} $$

Where **û** = ∑_j ≠ i_**Z**_j_*ĝ*_*j*_ and PEV is the predicted error variance.

Substituting *P*_*ik*_ = E(*b*_*ik*_|**y**) and using the above E_**û**_(**Q**), the expectation of Equation (6) over **û**, **b** is:

7 $$ \begin{array}{l}{\mathrm{E}}_{{\mathbf{b}}_{\boldsymbol{i}},\mathbf{u}\Big|\mathbf{y}} log p\left({g}_i,\mathbf{u},{\mathbf{b}}_{\boldsymbol{i}}\Big|\mathbf{y},\widehat{\mu},\kern0.75em \widehat{\sigma_e^2},\ \widehat{\mathbf{Pr}}\right)\\ {}=-\frac{n}{2} log\widehat{\sigma_e^2}-\frac{{\left({\mathbf{y}}^{*}-\widehat{\mathbf{u}}-{\mathbf{Z}}_{\mathrm{i}}{g}_i\right)}^{\hbox{'}}\left({\mathbf{y}}^{*}-\widehat{\mathbf{u}}-{\mathbf{Z}}_{\mathrm{i}}{g}_i\right)+\mathrm{t}\mathrm{r}\left(\mathrm{P}\mathrm{E}\mathrm{V}\left(\widehat{\mathbf{u}}\right)\right)}{2\widehat{\sigma_e^2}}\\ {}+{P}_{i1} log\left(\delta \left({g}_i\right){\widehat{Pr}}_1\right)+{\displaystyle \sum_{k=2}^4}{P}_{ik}\left[ log{\widehat{Pr}}_k-0.5* log{\sigma}_k^2-\frac{g_i^2}{2{\sigma}_k^2}\right]\ \\ {} + constant.\end{array} $$

The calculation of PEV(**û**) is approximated from a GBLUP model, and is explained in Additional file 2.

The M-step of emBayesR involved estimation of the SNP effect0073 (*g*_*i*_). Differentiating Equation (7) with regard to *g*_*i*_ gives:

$$ \begin{array}{l}\frac{\partial {\mathrm{E}}_{{\mathbf{b}}_{\boldsymbol{i}},\mathbf{u}\Big|\mathbf{y}} logp\left({g}_i,\mathbf{u},{\mathbf{b}}_{\boldsymbol{i}}\Big|\mathbf{y},\widehat{\mu},\kern0.75em \widehat{\sigma_e^2},\ \widehat{\mathbf{Pr}}\right)}{\partial {g}_i}\\ {}=\left[-{\displaystyle \sum_{k=2}^4}\frac{P_{ik}}{\sigma_k^2}-\frac{{\mathbf{Z}}_{\mathrm{i}}^{\hbox{'}}{\mathbf{Z}}_{\mathbf{i}}}{\widehat{\sigma_e^2}}\right]{g}_i+\frac{{\mathbf{Z}}^{\hbox{'}}\left(\mathbf{y}-\widehat{\mathbf{u}}-{\mathbf{1}}_{\boldsymbol{n}}\widehat{\mu}\right)}{\widehat{\sigma_e^2}}=0.\end{array} $$

Setting this equal to 0 results in the following posterior mode estimate for each SNP effect (*g*_*i*_).

8a $$ {\widehat{g}}_i={\left[{\mathbf{Z}}_{\mathrm{i}}^{\hbox{'}}{\mathbf{Z}}_{\mathbf{i}}+\left({P}_{i2}\frac{\widehat{\sigma_e^2}}{\sigma_2^2}+{P}_{i3}\frac{\widehat{\sigma_e^2}}{\upsigma_3^2}+{P}_{i4}\frac{\widehat{\sigma_e^2}}{\sigma_4^2}\right)\right]}^{-1}\left[{\mathbf{Z}}^{\hbox{'}}{\mathbf{y}}^{\dagger}\right], $$

where, **Z**_i_ is the *i*^*th*^ column of matrix **Z**, and ${\mathbf{y}}^{\dagger}\kern0.5em =\kern0.5em \mathbf{y}-\widehat{\mathbf{u}}-{\mathbf{1}}_{\boldsymbol{n}}\widehat{\mu}$.

The mean of the posterior distribution can also be calculated as follows:

$$ \mathrm{E}\left(p\left({g}_i\Big|\mathbf{y},P{r}_k\right)\right)=\frac{{\displaystyle {\int}_{-\infty}^{+\infty }}\Big({\displaystyle {\sum}_{k=1}^4}{P}_{ik}p\left({g}_i\Big|{b}_{ik}=1,\mathbf{y}, \Pr \right){g}_i\mathrm{d}{g}_i}{{\displaystyle {\int}_{-\infty}^{+\infty }}\Big({\displaystyle {\sum}_{k=1}^4}{P}_{ik}p\left({g}_i\Big|{b}_{ik}=1,\mathbf{y}, \Pr \right)\mathrm{d}{g}_i}, $$

which reduces to:

8b $$ {\overline{g}}_i={\sum}_{k=1}^4{P}_{ik}{\left[\left({\mathbf{Z}}_{\mathrm{i}}^{\hbox{'}}{\mathbf{Z}}_{\mathbf{i}}+\frac{\sigma_e^2}{\sigma_k^2}\right)\right]}^{-1}\left[{\mathbf{Z}}^{\hbox{'}}{\mathbf{y}}^{\dagger}\right]. $$

The mode estimation of SNP effects (Equation 8a) was implemented in our EM iterations, unless otherwise stated. The posterior mean of Equation (8b) was used in some cases to evaluate the accuracy of genomic prediction using either the mode or mean estimates of SNP effects. Furthermore, to investigate the degree of shrinkage, the least square estimate of the SNP effect was also calculated for some examples:

$$ {g}_i^{ls}={\left({\mathbf{Z}}_{\mathbf{i}}\boldsymbol{\hbox{'}}{\mathbf{Z}}_{\mathbf{i}}\right)}^{-1}{\mathbf{Z}}_{\mathbf{i}}\hbox{'}\left(\mathbf{y}-{\mathbf{1}}_{\boldsymbol{n}}\mu \right). $$

Similar EM steps used for estimating *ĝ*_*i*_ (but with different full models) are applied to estimate other parameters, including the proportion of SNP effects in each distribution (**Pr**), the error variance $\left({\sigma}_e^2\right)$, and the mean (*μ*).

To obtain $\widehat{\mathbf{Pr}}$, we return to the full model Equation (1) with all SNP effects (**g**) included. We introduce the missing variables **b**, so the full likelihood is:

$$ p\left(\mathbf{Pr},\mathbf{b}\Big|\mathbf{y},\mu \right)\propto p\left(\mathbf{y}\Big|\mathbf{b}\right)p\left(\mathbf{b}\Big|\mathbf{Pr}\right)p\left(\mathbf{Pr}\right), $$

Note that *p*(**y**|**b**) does not involve **Pr**, so when we differentiate with respect to **Pr**, this term drops out and can, therefore, be ignored, resulting in:

$$ \begin{array}{l}p\left(\mathbf{b}\Big|\mathbf{Pr}\right)={\prod}_{i=1}^n{\prod}_{k=1}^4{\left(P{r}_k\right)}^{b_{ik}}\\ {}p\left(\mathbf{Pr}\right)={\prod}_{k=1}^4P{r}_k,\\ {} logp\left(\mathbf{b}\Big|\mathbf{Pr}\right)={\sum}_{i=1}^n{\sum}_{k=1}^4{b}_{ik} logP{r}_k,\\ {} logp\left(\mathbf{Pr}\right)={\sum}_{k=1}^4 logP{r}_k,\kern0.5em \mathrm{and}\\ {}{\mathrm{E}}_{\mathbf{b}\Big|\mathbf{y}} logp\left(\mathbf{b}\Big|\mathbf{Pr}\right)={\sum}_{i=1}^n{\sum}_{k=1}^4{P}_{ik} logP{r}_k,\\ {}\mathrm{where}\kern0.5em {P}_{ik}=E\left({b}_{ik}\Big|\mathrm{y},\ P{r}_k\right).\end{array} $$

Then, considering that ${\sum}_{k=1}^4P{r}_k\kern0.5em =\kern0.5em 1$, we use Lagrange multiplier *λ* and differentiate with respect to *Pr*_*k*_. Given that **Pr** follows a Dirichlet distribution:

$$ \begin{array}{l}\frac{\partial {\mathrm{E}}_{\mathbf{b}\Big|\mathbf{y}} logp\left(\mathbf{g},\mathbf{Pr},{b}_{ik}\Big|\mathbf{y},\mu \right)+\uplambda \left({\displaystyle {\sum}_{k=1}^4}P{r}_k-1\right)\Big]}{\partial P{r}_k}\\ {}=\frac{{\displaystyle {\sum}_{i=1}^m}{P}_{ik}}{P{r}_k}+\frac{1}{P{r}_k}+\lambda =0.\end{array} $$

Therefore, the solution is:

9 $$ P{r}_k=\frac{{\displaystyle {\sum}_{i=1}^m}{P}_{ik}+1}{{\displaystyle {\sum}_{k=1}^4}\left({\displaystyle {\sum}_{i=1}^m}{P}_{ik}+1\right)}. $$

Finally, to estimate the error variance ${\sigma}_e^2$ and *μ*, we simplify Equation (5) into $\mathbf{y}={\mathbf{1}}_{\boldsymbol{n}}\mu +{\mathbf{u}}^{*}+\mathbf{e},\kern0.5em {\mathbf{u}}^{*}={\sum}_{\mathrm{i}=0}^m{\mathbf{Z}}_{\mathrm{i}}{\widehat{g}}_i$ and then the full likelihood based on this model is:

$$ \begin{array}{l}p\left({\sigma}_e^2,\mu, {\mathbf{u}}^{*}\Big|\mathbf{y}\right)=\\ {}\frac{1}{{\left(2\pi {\sigma}_e^2\right)}^{\frac{n}{2}}} exp\left[-\frac{1}{2{\sigma}_e^2}{\left(\mathbf{y}-{\mathbf{u}}^{*}-{\mathbf{1}}_{\boldsymbol{n}}\mu \right)}^{\hbox{'}}\left(\mathbf{y}-{\mathbf{u}}^{*}-{\mathbf{1}}_{\boldsymbol{n}}\mu \right)\right].\end{array} $$

The expectation for the full log likelihood based on this model is:

10 $$ \begin{array}{l}{\mathrm{E}}_{{\mathbf{u}}^{*}\Big|\mathbf{y}} logp\left({\sigma}_e^2,\mu, {\mathbf{u}}^{*}\Big|\mathbf{y}\right)\\ {}={\mathrm{E}}_{{\mathbf{u}}^{*}\Big|\mathbf{y}}\left[-\frac{n}{2}\mathrm{l}\mathrm{o}g{\sigma}_e^2+\frac{1}{2{\sigma}_e^2}{\left(\mathbf{y}-{\mathbf{u}}^{*}-{\mathbf{1}}_{\boldsymbol{n}}\mu \right)}^{\hbox{'}}\left(\mathbf{y}-{\mathbf{u}}^{*}-{\mathbf{1}}_{\boldsymbol{n}}\mu \right)\right]\\ {}=-\frac{n}{2}\mathrm{l}\mathrm{o}g{\sigma}_e^2+\frac{1}{2{\sigma}_e^2}\left[{\left(\mathbf{y}-\widehat{{\mathbf{u}}^{*}}-{\mathbf{1}}_{\boldsymbol{n}}\mu \right)}^{\hbox{'}}\left(\mathbf{y}-\widehat{{\mathbf{u}}^{*}}-{\mathbf{1}}_{\boldsymbol{n}}\mu \right)+tr\left(\mathrm{P}\mathrm{E}\mathrm{V}\left(\widehat{{\mathbf{u}}^{*}}\right)\right)\right].\end{array} $$

Therefore, differentiating Equation (10) with regard to ${\sigma}_e^2$ and *μ*, we get:

11 $$ {\sigma}_e^2=\frac{1}{n}\left[{\left(\mathbf{y}-\widehat{{\mathbf{u}}^{*}}-{\mathbf{1}}_{\boldsymbol{n}}\mu \right)}^{\hbox{'}}\left(\mathbf{y}-\widehat{{\mathbf{u}}^{*}}-{\mathbf{1}}_{\boldsymbol{n}}\mu \right)+\mathrm{t}\mathrm{r}\left(\mathrm{P}\mathrm{E}\mathrm{V}\left(\widehat{{\mathbf{u}}^{*}}\right)\right)\right], $$

12 $$ \mu =\frac{1}{n}{\left({\mathbf{1}}_{\boldsymbol{n}}\right)}^{\hbox{'}}\left(\mathbf{y}-\widehat{{\mathbf{u}}^{*}}\right) $$

for which computation of the term $\mathrm{t}\mathrm{r}\Big(\mathrm{P}\mathrm{E}\mathrm{V}\left(\widehat{{\mathbf{u}}^{*}}\right)$ is explained in Additional file 2.

In order to demonstrate the importance of the PEV correction for SNP effect estimates, the accuracy of emBayesR with and without accounting for PEV will be compared in the Results section. emBayesR without PEV has a similar EM step as emBayesR with PEV to derive the parameters ${P}_{ik},\kern0.5em {\widehat{g}}_i,\kern0.5em P{r}_k,\kern0.5em {\sigma}_e^2$ and *μ* but differs in the equations of emBayesR with PEV to calculate *P*_*ik*_ (Equation A3 in Additional file 1) and ${\sigma}_e^2$ (Equation 11) in that the term *tr*(PEV(**û**)) is not included in emBayesR without PEV.

### The emBayesR algorithm

The emBayesR algorithm can be described as follows:

#### Step 1

Initialise starting values for $\mathbf{g},\kern0.5em \mathbf{Pr},\kern0.5em {\sigma}_e^2,\kern0.5em {\sigma}_g^2,\kern0.5em \boldsymbol{\upalpha}$ and ${\boldsymbol{\upsigma}}_{\boldsymbol{k}}^2$. There are two groups of parameters: fixed parameters and changing parameters. $\boldsymbol{\upalpha} =\left(1,1,1,1\right),\kern0.5em {\sigma}_g^2$ and ${\boldsymbol{\upsigma}}_{\boldsymbol{k}}^2$ are fixed parameters, where **α** is the prior parameter for **Pr**, and ${\sigma}_g^2$ is used to set the value of ${\boldsymbol{\upsigma}}_{\boldsymbol{k}}^2$. The other variables $\left(\boldsymbol{g},\mathbf{Pr},{\sigma}_e^2\right)$ are updated during EM iterations. We used **g** = 0.01 and **Pr** = {0.5, 0.487, 0.01, 0.003}, as in [5]. To initialise ${\sigma}_e^2$ and ${\sigma}_g^2$, we used GBLUP implemented through ASREML3.0 [33] to estimate the error variance ${\sigma}_e^2$ and the genetic variance ${\sigma}_g^2$ as inputs for the next steps. Then, as mentioned before, the value of ${\sigma}_g^2$ defines ${\boldsymbol{\upsigma}}_{\boldsymbol{k}}^2$, using ${\boldsymbol{\upsigma}}_{\mathbf{k}}^2=\left\{0,\ 0.0001\kern0.5em *\kern0.5em {\sigma}_g^2,0.001\kern0.5em *\kern0.5em {\sigma}_g^2,0.01\kern0.5em *\kern0.5em {\sigma}_g^2\right\}$ for the real data and ${\boldsymbol{\upsigma}}_{\mathbf{k}}^2=\left\{0,\ 0.0006\kern0.5em *\kern0.5em {\sigma}_g^2,0.006\kern0.5em *\kern0.5em {\sigma}_g^2,0.06\kern0.5em *\kern0.5em {\sigma}_g^2\right\}$ for the simulated data.

#### Step 2

Calculate **PEV** with Equation (A7) of Additional file 2 (or it can be taken from ASREML in the step above).

Then for each SNP *i* (*i* in 1:m):

#### Step 3

Correct **y** for the effects of all other SNPs except the current SNP *i*, using:

$$ {\mathbf{y}}^{\dagger }=\mathbf{y}-{\sum}_{j\ne i}{\mathbf{Z}}_{\mathbf{j}}{\widehat{g}}_j-{\mathbf{1}}_{\boldsymbol{n}}\widehat{\mu} $$

#### Step 4

Estimate the probability that the effect of SNP *i* is from one of four normal distributions *logl*_*ik*_ with Equation (A5) of Additional file 1.

#### Step 5

Calculate *P*_*ik*_ with Equation (A6) of Additional file 1.

#### Step 6

Estimate the effect of SNP *i* with Equation (8a).

#### Step 7

After all SNP effects have been estimated, calculate *Pr*_*k*_ with Equation (9), update ${\sigma}_e^2$ with Equation (11), and update *μ* with Equation (12).

#### Step 8

Return to Step 3 and iterate until convergence. Here, the convergence criterion evaluated at each iteration *q* was (**ĝ**^*q*^ − **ĝ**^*q* − 1^) ' (**ĝ**^*q*^ − **ĝ**^*q* − 1^)/((**ĝ**^*q*'^**ĝ**^*q*^) < *γ*. The criterion *γ* = 10^− 10^ was selected after trialling the algorithm in a number of datasets and investigating changes in SNP effect estimates across iterations.

We calculated the time complexity of the algorithm (the function with parameters number of SNPs and number of animals that determines the time taken for the algorithm to run) based on the above eight steps. Time complexity is estimated in computer science applications by counting the number of innermost loops for elementary operations, which is notated *O*. For example, *O*(*n*) means the elementary operations in the algorithm need to be looped *n* times.

emBayesR need *q* loops to be converged. For each loop, Equation (A5) of Additional file 1 (Step 4 in the EM loop of emBayesR algorithm), is located in the innermost loop for the iteration. To be mentioned, both tr(PEV(**û**)) and $\mathrm{t}\mathrm{r}\left({\mathbf{Z}}_{\mathbf{i}}{\mathbf{Z}}_{\mathbf{i}}^{\hbox{'}}\mathrm{P}\mathrm{E}\mathrm{V}\left(\widehat{\mathbf{u}}\right)\right)$ in Equation (A5) are required, but fortunately they can be calculated outside EM iterations [See Additional file 1 for details]. Then, except for these two terms tr(PEV(**û**)) and $\mathrm{t}\mathrm{r}\left({\mathbf{Z}}_{\mathbf{i}}{\mathbf{Z}}_{\mathbf{i}}^{\hbox{'}}\mathrm{P}\mathrm{E}\mathrm{V}\left(\widehat{\mathbf{u}}\right)\right)$, the calculation number of Equation (A5) is the number of SNPs (*m*) × the number of animals (*n*). Therefore, the time complexity of each iteration in emBayesR is *O*(*mn*).

### Simulated data

Simulated data were used to determine how close the genomic prediction accuracy of emBayesR was to that of BayesR. The simulated dataset described in [21] was used. Briefly, FREGENE was used to simulate whole-genome sequence data in a population with an effective size (Ne) of 25 900 and a genome size of 50 Mb split equally over 10 chromosomes. The genome size of 50 Mb was chosen for computing efficiency. The accuracy of prediction in a c times larger genome (i.e. 50c Mb) would be approximately the same as found in our 50 Mb genome, provided the number of animals was c times larger than used here (i.e. 5000c) [27]. The mutation rate per bp was 9.38 × 10^−9^ and the recombination rate was 1 × 10^−8^ per base pair per generation [21], based on estimates for these rates in mammals. To ensure a drift-recombination-mutation equilibrium, the population was run for 370 000 generations. A total of 10 050 markers (including 50 QTL) were randomly selected as SNPs for genomic prediction. The SNP density was equivalent to ~600 000 SNPs on a 3000 Mb genome, similar to many mammals. Fifty QTL were randomly picked from the segregating loci, which is equivalent to 3000 QTL on a human or bovine genome. To evaluate the genomic prediction performance of emBayesR, BayesR and other algorithms, we generated two genetic architectures that differed in the distribution of true QTL effects. For this first dataset, named HD_Mix, the 50 QTL allele substitution effects were sampled from an equal mixture of three normal distributions with variances $\left(0,\ 0.0006{\sigma}_g^2,\ 0.006{\sigma}_g^2,\ 0.06{\sigma}_g^2\right)$. For the second genetic architecture (referred to as HD_One), QTL allele substitution effects were sampled from a single normal distribution. For the breeding values on simulation data, true breeding values (TBV) for individuals were obtained by summing genetic values across QTL. For each genetic architecture, heritabilities (*h*^*2*^) of either 0.45 or 0.1 were used. For each set, phenotypes of 5000 individuals were generated by adding a random residual value to the TBV of each individual. This residual value was sampled from a normal distribution, *N (0, σ*^*2*^_*e*_*)*, here *σ*^*2*^_*e*_ *=* [*σ*^*2*^_*TBV*_(*1-h*^*2*^)]/*h*^*2*^, where *σ*^*2*^_*TBV*_ is the variance of TBV in the population. Thus, we generated four datasets named HD_Mix_45 (five replicates following the mixture data model with heritability 0.45), HD_Mix_10 (five replicates following the mixture data model with heritability 0.10), HD_One_45 (five replicates following the one normal data distribution with heritability 0.45) and HD_One_10 (five replicates following the one normal distribution with heritability 0.10). Each replicate entailed sampling new SNP effects and generating new phenotypes.

To compare prediction accuracies and computing efficiencies of emBayesR, BayesR, GBLUP and fastBayesB, 5000 individuals were randomly separated into reference sets and validation sets. With an *h*^*2*^ of 0.45, there were 2500 individuals in the reference set and 2500 in the validation set. With an *h*^*2*^ of 0.1, there were 3750 individuals in the reference set and 1250 in the validation set. Accuracies were the correlations between GEBV and TBV.

### Real data

A total of 3354 Holstein-Friesian bulls were genotyped for both the Illumina Bovine HD SNP array (632 003 SNPs following quality controls as described in [5]), and the Bovine SNP 50 array (43 025 SNPs). Bulls genotyped at the lower density were imputed to the higher density using Beagle 3.0 [34], and applying quality controls as described in [5]. Phenotypes were daughter trait deviations (DTD) from two groups of traits: functional traits, including angularity, mammary conformation, stature, fertility (calving interval) and somatic cell count (SCC), and production traits, including milk yield, protein yield, protein % and fat %. For some of these traits, known QTL with moderate to large effects segregate in this population, for example a mutation in the *DGAT1* gene affects fat % [35]. Bulls were split into reference and validation sets by age, with the youngest bulls in the validation set. The numbers of bulls in the reference and validation sets for each trait are listed in Table 1. As a surrogate for prediction accuracy, the correlation of GEBV and DTD in the validation set was used. To investigate the computing time required for emBayesR relative to BayesR with different numbers of SNPs, we also ran genomic predictions in the same data but with the 50 K SNP chip genotypes (38 968 SNPs) extracted from the 630 K data on 3354 animals, for milk yield.

**Table 1:** **Numbers of Holstein bulls in the reference and validation sets for functional traits and production traits**

	**Reference set**	**Validation set**
Milk	3049	262
Protein	3049	262
Fertility	2806	396
Protein%	3049	262
Fat%	3049	262
Angularity	1484	251
Mammary conformation	1484	251
Stature	1484	251
Somatic cell count	2662	410

## Results

The results are presented in three sections. First, we investigated the convergence of parameters estimated by emBayesR and how close parameter estimates from emBayesR were to the true parameter values, and those estimated by BayesR, in terms of SNP effects and **Pr**, in the simulated data. We also evaluated the effect of the PEV correction on estimates of these parameters, and the accuracy of genomic prediction. Moreover, the accuracy of genomic prediction from the joint posterior mode estimation from emBayesR was compared to the accuracy when the posterior mean estimate of SNP effects was used. The mode estimation for SNP effects (Equation 8a) of emBayesR was used for the evaluation of performance of emBayesR. Thus, we also compared the accuracy of prediction with mode (8a) and mean (8b) Equations for estimates of SNP effects (Equation 8b). In the second section of results, we compared the accuracy of genomic prediction from emBayesR to that of BayesR, as well as computing speed in simulated and real datasets. Finally, the sensitivity of prediction accuracy from emBayesR to the underlying genetic architecture (multi-normal distribution, normal distribution of QTL effects, real 630 K data) was investigated.

### Convergence of parameter estimates with emBayesR

**Figure 1:**
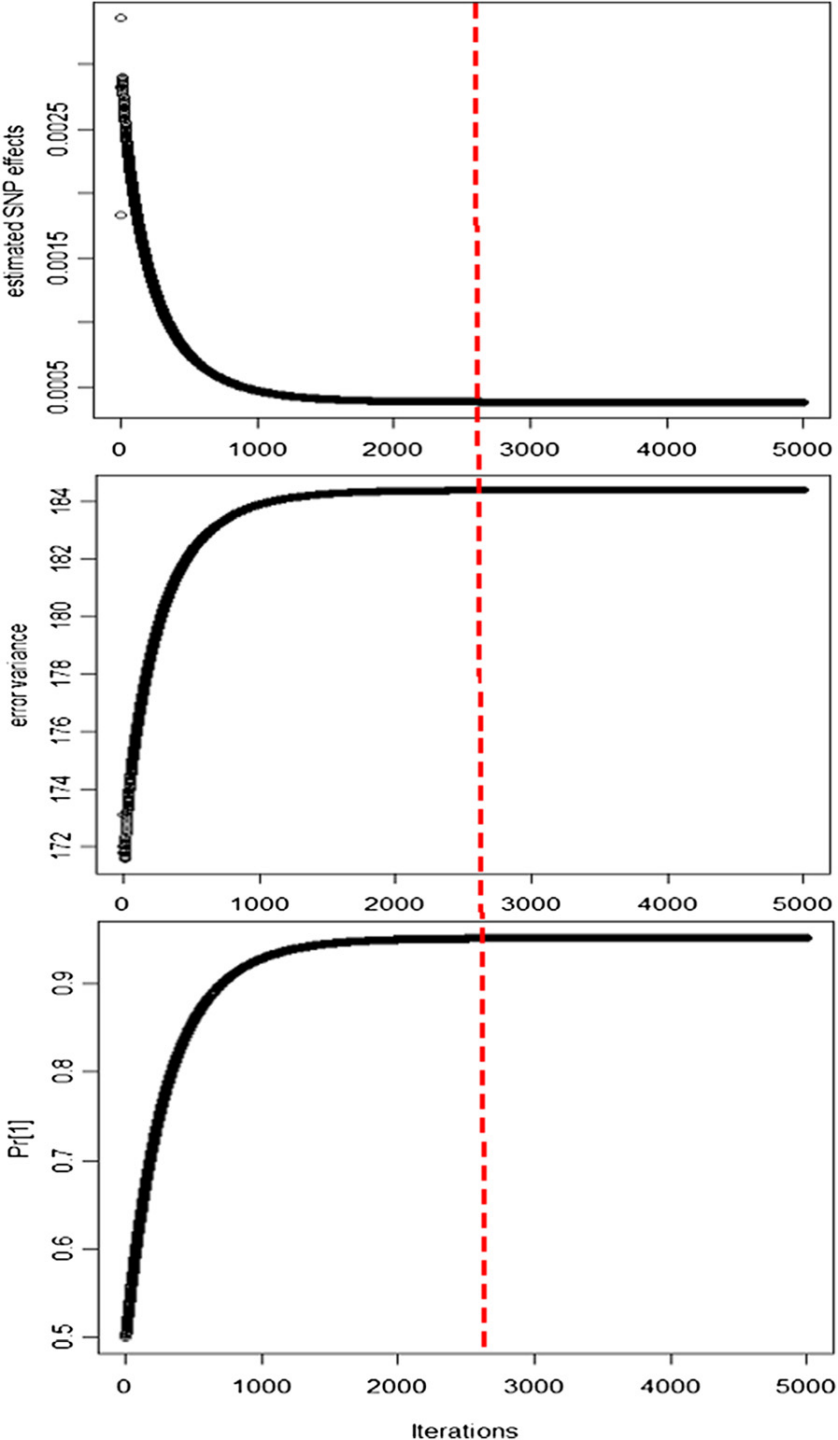
**Convergence of estimated SNP effects, error variance and Pr over 5000 iterations.** The x axis represents the number of iterations that range from 0 to 5000; the y axis represents the estimated SNP effects, error variance and the first element of Pr (the proportion of SNPs in the distribution with zero variance).

The algorithm is considered to have “converged” when estimated SNP effects from the previous iteration are very close to estimated SNP effects in the current iteration. The convergence criterion of emBayesR was (**ĝ**^*q*^ − **ĝ**^*q* − 1^) ' (**ĝ**^*q*^ − **ĝ**^*q* − 1^)/((**ĝ**^*q*'^**ĝ**^*q*^) < 10^− 10^, where *q* is the current iteration number. Since the convergence criterion assessed only changes in SNP effect estimates, it does not guarantee that the estimates of the other parameters, i.e. **Pr** (the proportion of SNPs in each distribution) and the error variance, have converged. In the simulated dataset HD_Mix_45, convergence was reached after 2500 iterations, and at that point, there was also very little change in the error variance and **Pr** from the previous iteration (Figure 1).

### Comparison of parameter estimates

**Figure 2:**
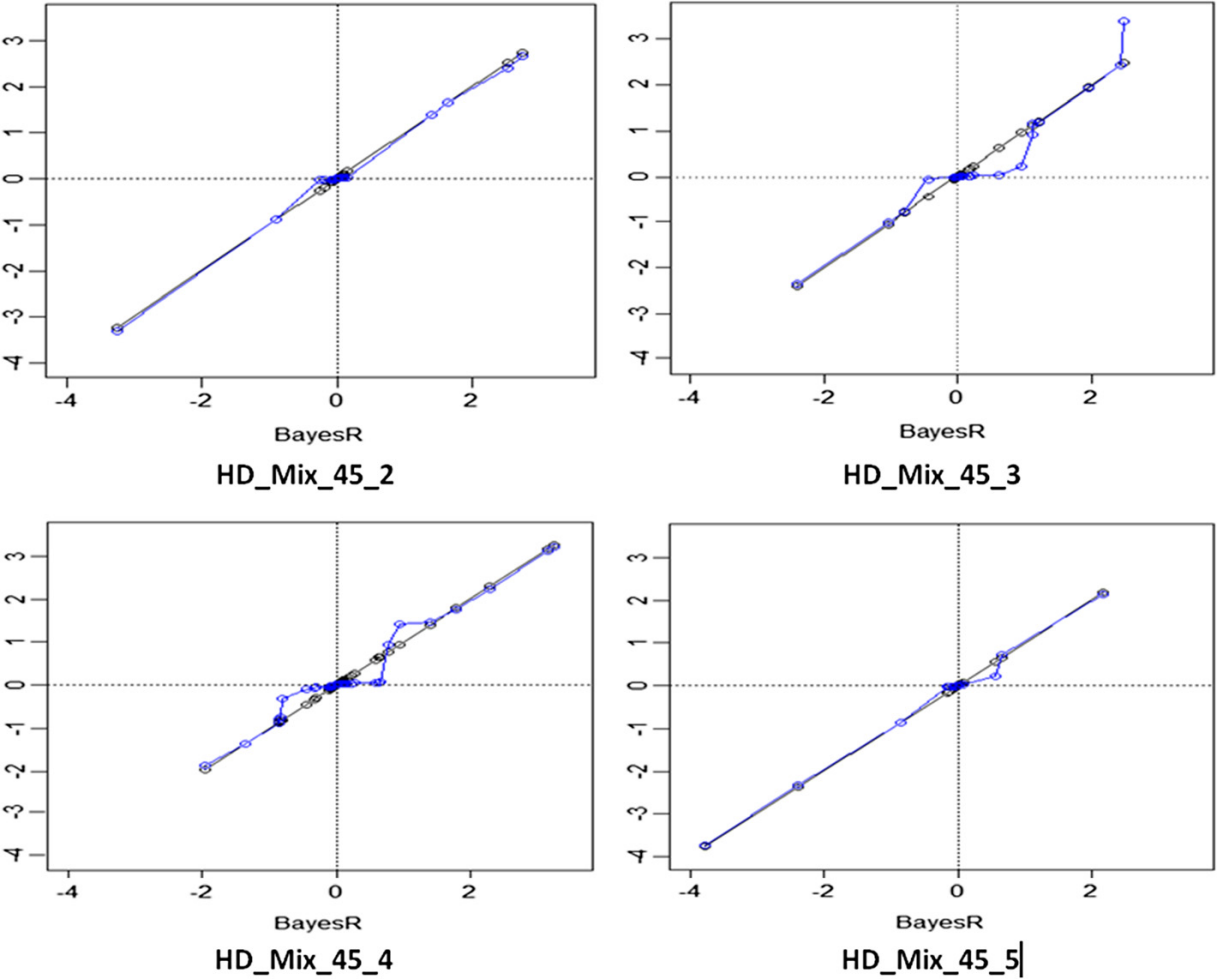
**Correlation between SNP effects from BayesR and emBayesR SNP effects in four replicates of HD_Mix_45 (h** ^**2**^ **= 0.45).** The x axis represents the BayesR estimates of SNP effect; blue line plots emBayesR estimates of SNP effects on BayesR estimates of SNP effects; black line plots BayesR estimates of SNP effects on themselves for four replicates of HD_Mix with a heritability of 0.45.

**Figure 3:**
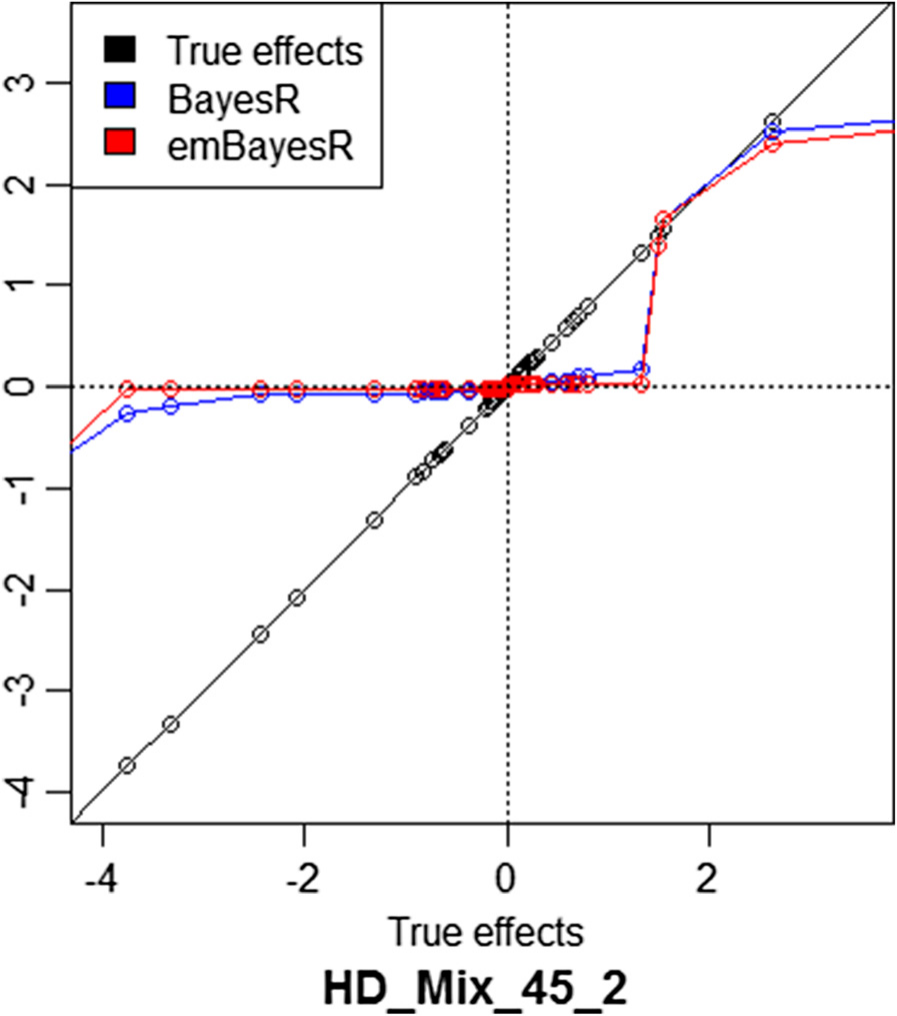
**Estimates of SNP effects from BayesR and emBayesR compared with their true effects in one replicate of HD_Mix_45 (HD_Mix_45_2).** The x axis represents true effects; blue curve plots BayesR estimates of SNP effects on true effects; red line plots emBayesR estimates of SNP effects on true effects; the black line plots true effects on themselves for one replicate of simulated data HD_Mix with a heritability of 0.45 (HD_Mix_45_2).

Estimates of SNP effects and **Pr** from emBayesR can be compared to the corresponding estimates from BayesR. For the HD_Mix simulated data, estimates of large SNP effects were very similar for BayesR and emBayesR (Figure 2). The plot of BayesR and emBayesR estimated effects against true effects is in Figure 3. However, for smaller effects, emBayesR shrunk effects to a greater degree than BayesR, in some replicates.

**Figure 4:**
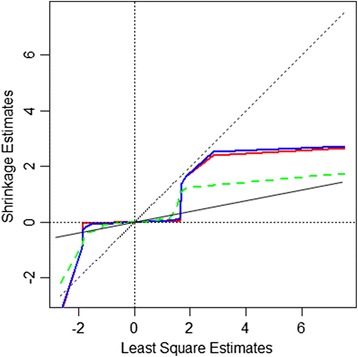
**Estimates of SNP effects from SNP-BLUP, BayesR, emBayesR, FastBayesB against their least square estimates.** The x axis represents the least square estimates of SNP effects; blue line plots BayesR estimates of SNP effects on the least square estimates; red line represents emBayesR SNP effect estimates; dotted green line represents the fastBayesB estimates of SNP effects; black line represents SNP_BLUP estimates of SNP effects for HD_Mix_45.

The degree of shrinkage from the BayesR algorithms relative to other algorithms can be demonstrated by plotting estimates of SNP effects (HD_Mix data set) from BayesR, FastBayesB, emBayesR and SNP-BLUP against their least square estimates (Figure 4). Both BayesR and emBayesR regressed moderate size SNP effects towards 0 more than SNP-BLUP and FastBayesB. However, BayesR and emBayesR did not shrink large SNP effects nearly as much as SNP-BLUP.

Estimates of **Pr** from emBayesR and BayesR are compared with the true proportion of SNP effects in each of the four normal distributions in Table 2. The genetic architecture of the HD_Mix data was such that 50 QTL were distributed evenly in three normal distributions with non-zero variances. The true proportion of the SNP effects (around 10 000 markers) in the four normal distributions with different variances $\left(0,\ 0.0006{\sigma}_g^2,\ 0.006{\sigma}_g^2,\ 0.06{\sigma}_g^2\right)$ was (0.995, 0.0017, 0.0016, 0.0017). As shown in Table 2, when *h*^*2*^ = 0.45, both BayesR and emBayesR estimated the proportions of SNP effects from the four distributions to be roughly 0.99, 0.01, 0.001, and 0.001. However, when *h*^*2*^ = 0.1, BayesR over-estimated the proportion of SNP effects in the smallest non-zero distribution $\left({\sigma}_2^2 = 0.0006{\sigma}_g^2\right)$ and this tendency was even greater with emBayesR. This agrees with results in Figure 2, where emBayesR shrunk small effects to very small effects more than BayesR and this may have contributed to the over-estimation of the proportion of SNP effects from the distribution with the smallest non-zero variance $\left(0.0006{\sigma}_g^2\right)$. In the 630 K dairy cattle data, the posterior mean estimates of **Pr** from emBayesR were similar to those from BayesR, as shown in Table 3.

**Table 2:** **Estimated mixing proportions (Pr) from BayesR and emBayesR in the 10 k simulation data (HD_Mix_45)**

**Five replicates of 10 K simulation data with h** ^**2**^ **= 0.45**		
**True value of Pr [0.9950 0.0017 0.0016 0.0017]**		
	**BayesR**	**emBayesR**
**M45_1**	[0.9865 0.0110 0.0010 0.0015]	[0.9813 0.0163 0.0009 0.0015]
**M45_2**	[0.9861 0.0127 0.0004 0.0008]	[0.9852 0.0136 0.0003 0.0009]
**M45_3**	[0.9933 0.0046 0.0009 0.0012]	[0.9899 0.0083 0.0005 0.0012]
**M45_4**	[0.9909 0.0055 0.0022 0.0015]	[0.9864 0.0110 0.0010 0.0016]
**M45_5**	[0.9944 0.0043 0.0006 0.0007]	[0.9910 0.0078 0.0005 0.0007]
**Five replicates of 10 K simulation data with h** ^**2**^ **= 0.10**		
**True value of Pr [0.9950 0.0017 0.0016 0.0017]**		
	**BayesR**	**emBayesR**
**M10_1**	[0.9759 0.0021 0.0024 0.0010]	**[0.9243 0.0741 0.0009 0.0008]**
**M10_2**	[0.9624 0.0343 0.0025 0.0009]	**[0.9086 0.0898 0.0010 0.0007]**
**M10_3**	[0.9757 0.0022 0.0018 0.0008]	**[0.9284 0.0702 0.0007 0.0007]**
**M10_4**	[0.9620 0.0334 0.0032 0.0014]	**[0.9146 0.0837 0.0008 0.0010]**
**M10_5**	[0.9664 0.0295 0.0023 0.0018]	**[0.9265 0.0715 0.0007 0.0014]**

**Table 3:** **Estimated mixing proportions (Pr) from BayesR and emBayesR for the 630 k real dairy cattle data**

	**BayesR**	**emBayesR**
Milk	[0.99291 0.00690 0.00018 0.00001]	**[0.99511 0.00480 0.00006 0.00003]**
Protein	[0.99161 0.00831 0.00005 0.00003]	**[0.99480 0.00511 0.00007 0.00002]**
Fertility	[0.98863 0.01034 0.00092 0.00011]	**[0.99184 0.00806 0.00009 0.00001]**
Protein%	[0.99602 0.00378 0.00019 0.00001]	**[0.99902 0.00078 0.00004 0.00016]**
Fat%	[0.99480 0.00485 0.00021 0.00014]	**[0.99786 0.00204 0.00001 0.00009]**
Angularity	[0.99221 0.00739 0.00039 0.00001]	**[0.98514 0.01475 0.00009 0.00002]**
Mammary conformation	[0.99091 0.00859 0.00047 0.00003]	**[0.99276 0.00714 0.00009 0.00001]**
Stature	[0.99013 0.00927 0.00052 0.00008]	**[0.99305 0.00684 0.00006 0.00005]**
Somatic cell count	[0.98688 0.01272 0.00039 0.00001]	**[0.98761 0.01229 0.00008 0.00002]**

### Sensitivity to the prior for the Dirichlet distribution

Another feature of estimates of **Pr**, may be sensitivity to its prior parameter ***α*** (the pseudo-count of SNPs in each distribution in the Dirichlet distribution). To evaluate the sensitivity of emBayesR to ***α***, we used different values for ***α*** and investigated the effect on **Pr** with the dataset HD_Mix_45 (Table 4). When the prior parameter ***α*** was changed from (1, 1, 1, 1) to (100, 1, 1, 1), estimates of **Pr** from emBayesR changed only slightly. Although **α** = (100, 1, 1, 1) was closer to the true situation in the simulated datasets, estimates for **Pr** (especially *Pr*[2], *Pr*[3], *Pr*[4]) deviated from the true values [0.9950 0.0017 0.0016 0.0017]. When ***α*** was changed to (1, 1, 1, 100) and (1, 1, 100, 1), the estimate of **Pr** was affected, with the proportion of SNP effects estimated to be in the distribution with α[4] = 100 increasing to 0.0027 and 0.0028, respectively, instead of the simulated 0.0017. It is not surprising that a pseudo-count of 100 affected the estimate of **Pr**, since the true number of SNP effects in these distributions was equal to 17 only. Interestingly, the prediction accuracy remained at 0.97 in spite of these changes in the prior **α**.

**Table 4:** **Pr estimates (proportion of SNP in each distribution) with different prior values α for the HD_Mix_45 simulated data**

**α**	**Pr_emBayesR**			
	**0**	$\mathbf{0.0006}\kern0.5em *\kern0.5em {\boldsymbol{\upsigma}}_{\mathbf{g}}^{\mathbf{2}}$	$\mathbf{0.006}\kern0.5em *\kern0.5em {\boldsymbol{\upsigma}}_{\mathbf{g}}^{\mathbf{2}}$	$\mathbf{0.06}\kern0.5em *\kern0.5em {\boldsymbol{\upsigma}}_{\mathbf{g}}^{\mathbf{2}}$
(1, 1, 1, 1)	0.9861	0.0127	0.0004	0.0008
(1, 1, 1, 100)	0.9801	0.0130	0.0042	0.0027
(1, 1, 100, 1)	0.9863	0.0101	0.0028	0.0008
(100,1, 1, 1)	0.9883	0.0105	0.0003	0.0009

The prior **α** was (1, 1, 1, 1), (1, 1, 1, 100), (1, 100, 1, 1) or (100, 1, 1, 1).

### Effect of PEV

**Figure 5:**
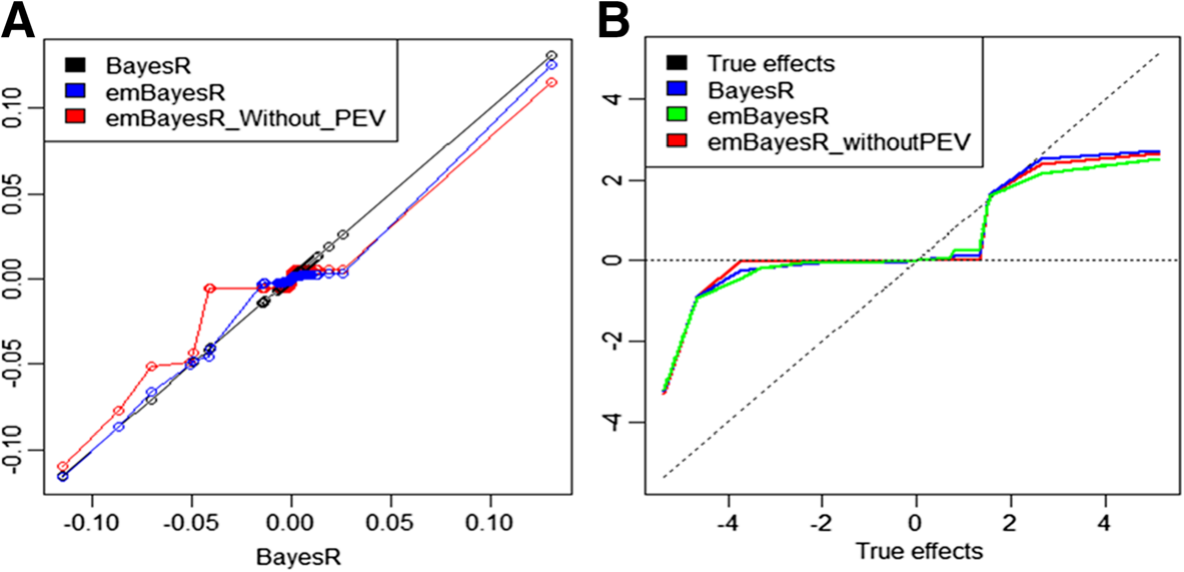
**Comparison of SNP effect estimates from emBayesR with and without accounting for PEV with estimates from BayesR. A**: The x axis represents BayesR estimates of SNP effects; blue line plots emBayesR estimates of SNP effects on BayesR estimates of SNP effects; red line plots emBayesR_Without_PEV estimates of SNP effect on BayesR estimates of SNP effects; black line plots BayesR estimates of SNP effects against themselves. **B**: The x axis represents true effects; blue line plots BayesR estimates of SNP effects on true effects; green line plots emBayesR estimates of SNP effects on true effect; red line plots emBayesR_without_PEV estimates of SNP effects on true effects; black line plots true effects against themselves.

We also compared estimates of parameters and accuracies of genomic prediction with and without accounting for PEV or estimates of all other SNPs in the emBayesR algorithm. When the PEV was accounted for in the emBayesR algorithm, there was a 6% improvement in the accuracy of genomic prediction in the simulated data when *h*^*2*^ = 0.45, and 5% when *h*^*2*^ = 0.1 (Table 5), compared to when PEV was not accounted for. Estimates of SNP effects from emBayesR with and without PEV were plotted against estimates of SNP effects from BayesR (Figure 5A). Estimates of SNP effects from emBayesR without accounting for PEV were considerably shrunken, particularly for small effects, compared with estimates of SNP effect from BayesR. Estimates of SNP effects with emBayesR when PEV were accounted for were much closer to those from BayesR, although there was still some over-shrinkage, particularly of small effects. Figure 5B, in which estimates of SNP effects obtained with BayesR, emBayesR, emBayesR_without_PEV are plotted, illustrates this result.

**Table 5:** **Accuracy of genomic prediction from emBayesR_without_PEV and emBayesR on HD_Mix dataset**

	**Correlation (GEBV,TBV)**				
**Five replicates with h** ^**2**^ **= 0.45 (HD_Mix_45)**	Rep 1	Rep 2	Rep 3	Rep 4	Rep 5
emBayesR_without_PEV	0.91	0.90	0.85	0.90	0.91
emBayesR	0.97	0.96	0.93	0.97	0.97
**Five replicates with h** ^**2**^ **= 0.10 (HD_Mix_10)**	Rep 1	Rep 2	Rep 3	Rep 4	Rep 5
emBayesR_without_PEV	0.89	0.82	0.87	0.81	0.79
emBayesR	0.91	0.87	0.93	0.86	0.87

We also compared the accuracy of prediction based on the joint posterior mean (Equation 8b) versus the mode (Equation 8a) in the simulated data (Table 6). As shown in Table 6, using either the mean (emBayesR_Mean) or the mode (emBayesR_Mode) for estimates of SNP effect gave similar prediction accuracies.

**Table 6:** **Accuracy of genomic prediction using the algorithm posterior mode (emBayesR_Mode, Equation 8a) or posterior mean estimates of SNP effects (emBayesR_Mean, Equation 8b), in the HD_Mix dataset**

	**Correlation (GEBV,TBV)**				
**Five replicates with h** ^**2**^ **= 0.45**	Rep 1	Rep 2	Rep 3	Rep 4	Rep 5
emBayesR_Mode	0.97	0.96	0.93	0.97	0.97
emBayesR_Mean	0.97	0.95	0.93	0.97	0.97
**Five replicates with h** ^**2**^ **= 0.10**	Rep 1	Rep 2	Rep 3	Rep 4	Rep 5
emBayesR_Mode	0.91	0.87	0.93	0.86	0.87
emBayesR_Mean	0.91	0.88	0.93	0.87	0.87

### Accuracy of genomic prediction with emBayesR and BayesR

In the simulation data, the accuracy of genomic prediction with emBayesR was the same as with BayesR when heritability was 0.10, but 1% lower when heritability was 0.45 (Table 7). However, both methods resulted in GEBV that were close to unbiased, based on the regression of TBV on GEBV being close to 1, although for HD_Mix_10, the regression was 0.89 with both BayesR and emBayesR.

**Table 7:** **Accuracy of genomic prediction and the regression coefficient of true breeding value (TBV) on genomic estimated breeding value (GEBV) for different methods for the HD_Mix simulated dataset**

	**Correlation (GEBV,TBV)**		**Regression coefficient (TBV on GEBV)**	
	**h2 = 0.45**	**h2 = 0.10**	**h2 = 0.45**	**h2 = 0.10**
	**2500 animals**	**3750 animals**	**2500 animals**	**3750 animals**
BayesR	0.97 ± 0.01	0.89 ± 0.03	1.02 ± 0.02	1.00 ± 0.05
emBayesR	**0.96** ± **0.03**	**0.89** ± **0.02**	**0.95** ± **0.03**	**1.00** ± **0.04**

Accuracies of genomic prediction with BayesR, GBLUP, FastBayesB, and emBayesR on the 630 K dairy data are in Table 8. The average accuracy of genomic prediction with emBayesR across the nine dairy cattle traits was 0.4% lower than with BayesR. The accuracy with emBayesR was on average 5% better than with FastBayesB. The average accuracy of BayesR across the nine traits was 3% higher than with GBLUP, which was due to very similar accuracies for four of the nine traits, and only protein % and fat % showing clear improvements in accuracy compared to GBLUP. For these traits, several QTL with moderate to large effects are known to exist [35,36].

**Table 8:** **Accuracy of genomic prediction from GBLUP, BayesR, fastBayesB and emBayesR for the 630 K dairy cattle data for production and functional traits**

	**Production traits**				
	**Milk**	**Protein**	**Fertility**	**Protein%**	**Fat%**
GBLUP	0.57	0.63	0.40	0.63	0.77
BayesR	0.63	0.64	0.41	0.79	0.83
FastBayesB	0.57	0.60	0.35	0.70	0.80
emBayesR	**0.62**	**0.65**	**0.40**	**0.76**	**0.83**
	**Functional traits**				
	**Angularity**	**Mammary conformation**	**Stature**	**Somatic cell count**	
GBLUP	0.45	0.28	0.47	0.71	
BayesR	0.44	0.28	0.47	0.71	
FastBayesB	0.39	0.25	0.43	0.61	
emBayesR	**0.45**	**0.30**	**0.47**	**0.69**	

### Computing performance of emBayesR compared with BayesR

We compared the speed of emBayesR with BayesR and fastBayesB using three criteria: the time complexity of each iteration (the function in terms of number of SNPs and individuals that determines the time taken to do one iteration), the number of iterations to convergence (or in the case of BayesR until changes in SNP estimates were sufficiently small so that the accuracy of genomic prediction did not change), and total computing time required with the 630 K real data.

First, as mentioned in the method section, the time complexity for emBayesR is *O*(*nm*), which is the same as with the MCMC method for BayesR and with ICE iterations for fastBayesB, and with the nonlinear A method of VanRaden [2] and SNP_BLUP [1].

**Figure 6:**
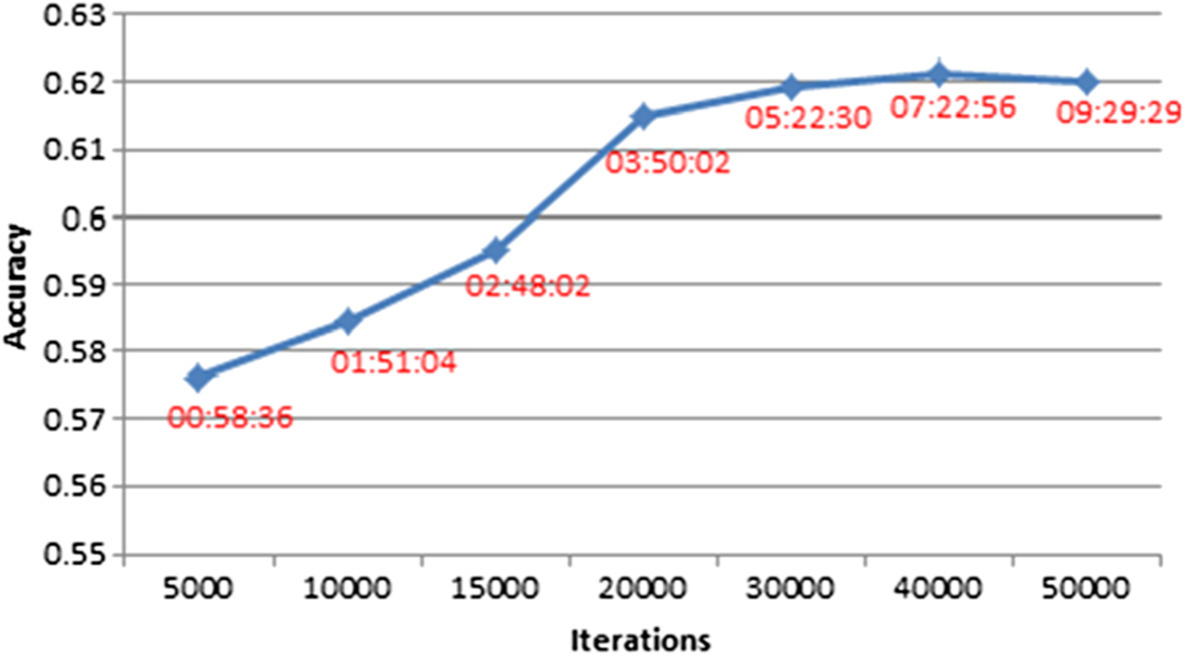
**Accuracy of genomic prediction and running time for BayesR with an increasing number of iterations.**

Second, for BayesR, the accuracy of prediction exceeded 0.61 at 20 000 iterations, and did not improve with a larger number of iterations, as shown in Figure 6. For five traits (milk, protein, fertility, fat % and protein %) and using the 630 K real data, the numbers of iterations required for convergence for emBayesR and fastBayesB are given in Table 9. FastBayesB required slightly more iterations to reach convergence than emBayesR for most traits.

**Table 9:** **Number of iterations required for emBayesR and fastBayesB to reach convergence for five traits with the 630 K dairy cattle data**

	**Milk**	**Protein**	**Fertility**	**Protein %**	**Fat %**
emBayesR	**460**	**476**	**920**	**572**	**496**
FastBayesB	410	540	856	848	564

**Figure 7:**
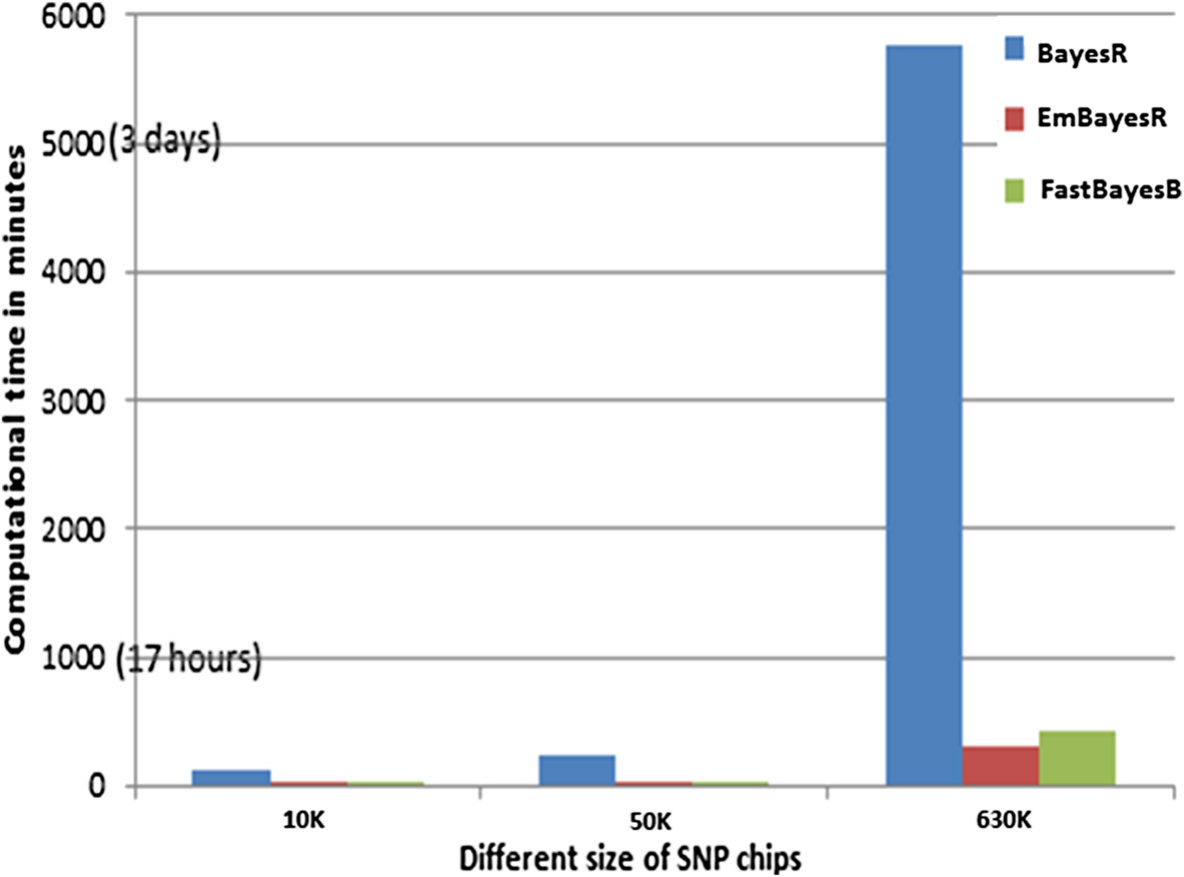
**Computational time required for BayesR, emBayesR and FastBayesB on a range of SNP chips (10 K, 50 K and 630 K).** The x axis represents the different sizes of the SNP chips, y axis is the computational time in minutes; blue bar is BayesR’s running time; red bar is emBayesR’s; green bar is FastBayesB’s computing time.

Finally, the overall computing times for emBayesR, BayesR and fastBayesB with the same implementation (each trait on one processor) were compared (Figure 7). The algorithms were implemented on a range of datasets with different sizes, including 10 K simulated data (HD_Mix model, 2500 animals with around 10 000 SNPs), 50 K data (3049 animals with 38 968 SNPs), and 630 K data (3049 animals with 632 003 SNPs). As shown in Figure 7, the speed advantage of emBayesR compared to BayesR was greater as the number of SNPs in the dataset increases. For example, with the 630 K data, BayesR needed approximately 4 days of real computing time, while emBayesR required just 4 hours (including the time to calculate PEV in GBLUP) to achieve the final solutions.

### Sensitivity of parameter estimates from emBayesR to the underlying genetic model

In this final Results section, we investigate the sensitivity of the accuracy of genomic prediction and estimates of **Pr** with emBayesR and BayesR to the underlying data model. Three underlying models for QTL effects were investigated: (1) an equal mixture of three non-zero normal distributions in HD_Mix; (2) all QTL effects follow a normal distribution in HD_One; and (3) an unknown model of QTL effects in the 630 K real data.

emBayesR and BayesR gave higher accuracies than GBLUP for the HD_Mix model data (M45_2), while for the HD_One data, the advantage of emBayesR and BayesR was smaller than that of GBLUP (Table 10), as might be expected given that the HD_Mix data has a proportion of QTL with larger effects. In estimating **Pr**, emBayesR generally had somewhat poorer agreement with the underlying data model than BayesR (Table 10), especially for the HD_One_45 data.

**Table 10:** **Estimated mixing proportions (Pr) and genomic prediction accuracy from BayesR, emBayesR and GBLUP with the HD_Mix_45 and HD_One_45 datasets**

**HD_Mix_45 (h** ^**2**^ **= 0.45)**		
	**Pr**	**Accuracy**
True	[0.9950 0.0017 0.0016 0.0017]	
BayesR	[0.9861 0.0127 0.0004 0.0008]	0.97
emBayesR	[0.9852 0.0136 0.0003 0.0009]	0.97
GBLUP	-	0.67
**HD_One_45 (h** ^**2**^ **= 0.45)**		
	**Pr**	**Accuracy**
True	[0 0 0 1]	
BayesR	[0.722 0.2621 0.0115 0.0044]	0.80
emBayesR	[0.012 0.986 0.0007 0.0013]	0.80
GBLUP	-	0.78

However, on 630 K real data, emBayesR gave very similar estimates of **Pr** and accuracy of genomic prediction than BayesR and GBLUP (accuracy only for the later comparison) (Tables 3 and 8). One conclusion from the relative performance of emBayesR to BayesR in the 10 K simulated data and in the 630 K real data, is that emBayesR cannot distinguish SNP effects with zero variance from those with a very small variance when there is little information in small datasets, as in the HD_One simulated data. However, among the 630 K SNPs there are likely more SNPs in the non-zero distributions, which should increase the precision of estimates of **Pr.**

## Discussion

Genomic prediction with non-linear Bayesian methods, including BayesR, can be more accurate than GBLUP in some situations, such as when QTL with moderate to large effects segregate [2,3], but at the cost of longer computing time. To retain the accuracy of BayesR while reducing computing time, we propose here an EM algorithm, termed emBayesR, for genomic prediction, as an alternative to the MCMC implementation of BayesR. In both 10 K SNP simulated data and 630 K real dairy cattle data, emBayesR gave accuracies of genomic prediction similar to BayesR, with greatly reduced computing time. As in BayesR, emBayesR estimates SNP effects, error variances and posterior probabilities of each SNP belonging to the *k*^*th*^ distribution (here, there were four distributions, one with zero variance).

Results from BayesR and emBayesR differed in three ways, albeit to a small degree. Estimates of **Pr** with emBayesR tended to have more SNP effect estimates in the smallest non-zero distribution than BayesR; emBayesR shrunk small SNP effects towards 0 somewhat more than BayesR; and the accuracy of emBayesR predictions was approximately 0.5% lower than the accuracy of BayesR. Our EM algorithm differed from the MCMC BayesR in several respects, which may explain these results. The EM algorithm estimates the SNP effect (*g*_*i*_) by the mode of the posterior distribution when the mixing proportions (**Pr**) and the error variance $\left({\sigma}_e^2\right)$ are held at their MAP estimates, whereas the MCMC version estimates *g*_*i*_ by the mean of the posterior distribution while **Pr** and ${\sigma}_e^2$ vary over their posterior distributions. Also, when we used the mean instead of the mode of the posterior distribution of *g*_*i*_ as an estimate of *g*_*i*_, we found that it makes no discernible difference in prediction accuracy, as shown in Table 6. However, varying **Pr** and ${\sigma}_e^2$ across their posterior distributions in BayesR, but not emBayesR, may explain differences in results. In addition, emBayesR uses an approximation of the prediction error variance of all other SNPs when estimating *g*_*i*_.

Bayesian estimates are sensitive to the prior if the data does not contain enough information to overwhelm the prior. Estimates of **Pr** with both BayesR and emBayesR were affected by the prior **α** but not to a large degree, considering that the simulated data contained only 50 causal mutations and the prior had little effect on the accuracy of genomic predictions. Results from using emBayesR with the simulated data indicate the algorithm was unable to consistently distinguish a SNP with no effect from a SNP with a very small effect. We would expect that, in data in which more causal mutations are segregating and with many more animals, estimates of **Pr** would be less sensitive to the prior.

Other EM algorithms for genomic prediction have been described using thick-tailed *t*-distributions or exponential distributions as priors for the SNP effects. These include EM-BSR [37] and FastBayesA [38], which aim at enhancing the computing efficiency of BayesA. emBayesR differs from most previous non-MCMC implementations of Bayesian methods for genomic prediction in two respects, i.e. it uses the BayesR model with a mixture of four normal distributions for SNP effects and it accounts for errors in all other estimated SNP effects when estimating the effect of the current SNP by including the PEV term in the model. When we implemented the EM algorithm without the PEV term, the accuracy of prediction declined by 8%. The accuracy of fastBayesB was, on average, 9% lower than that of emBayesR, suggesting that much of the loss in accuracy of fastBayesB is due to ignoring the errors in all other SNP effects when estimating a particular SNP effect. Consistent with this interpretation, both fastBayesB and our EM algorithm without accounting for the PEV shrink estimates of SNP effects more severely than emBayesR or BayesR. Most of the current fast algorithms, such as fastBayesB [29], emBayesB [31], em_BSR [37], and MixP [39], ignore the error produced by the estimation of other SNP effects. That is, they use an unrealistic assumption that the current solutions of all other SNPs effects are known without error when estimating the current SNP effect, which is one of the reasons why accuracies of prediction from these algorithms are typically lower than that of their counterpart MCMC methods. MCMC methods account for the error in the estimates of other SNP effects by sampling them from their posterior distributions. For the calculation of PEV, the inverse of a matrix with dimensions (number of animals × the number of animals) is required (Equation (A7) of Additional file 2). When the number of animals exceeds 50 000, this will hinder the computing efficiency of emBayesR. To reduce the computing burden of the PEV calculation, the efficient genomic recursion algorithms proposed by Misztal et al. [13] could be applied but this requires further investigation.

Our results demonstrated the computing speed of emBayesR over the MCMC implementation of BayesR. The time complexity for emBayesR at each iteration is proportional to the number of markers and the number of records, as it is in the MCMC methods. However, much fewer iterations were required for the emBayesR SNP effects to converge than for BayesR to sample sufficiently from the posterior distributions of SNP effects to achieve maximum accuracy of genomic prediction. Specifically, compared with 20 000 iterations of MCMC sampling (Figure 6), emBayesR required only 300 to 1000 iterations with the 630 K real dairy data (Table 9). As the size of datasets increased, this advantage could be even greater, as shown in Figure 7.

With high-density SNP data (630 K), the prediction accuracy of emBayesR and BayesR was greater than GBLUP only for yield traits. Similar results (an advantage of a Bayesian approach over GBLUP for yield traits only) were obtained using the nonlinear iterative A method with imputed high-density data from 15 842 reference animals and 28 traits [40]. Computing time with high-density data for this nonlinear A method is also *O*(*nm*), with reported times similar to emBayesR. One difference between BayesR and the nonlinear A method is that SNP effects can actually be 0 with BayesR, whereas in nonlinear A, SNPs will always have a non-zero effect, although it may be very small. This difference between the algorithms apparently does not affect accuracies of prediction with the 630 K real data, although it may become more important with whole-genome sequence data, for which the number of variants is much larger. However, this is yet to be demonstrated.

It should also be noted that some reduction in computing time can be achieved by “pruning” SNPs that are in very high linkage disequilibrium from the dataset, since these SNPs carry redundant information. For example, Su et al. [41] reduced a dataset from 770 K to 492 K SNPs by pruning SNPs that were in very high linkage disequilibrium in a Nordic Holstein population prior to estimation of SNP effects.

Our aim is to eventually integrate emBayesR into genetic evaluations for Australian dairy cattle. Currently, the Australian National DNA reference population has more than 20 000 cattle, including 3719 Holstein bulls, 9630 Holstein cows, 1017 Jersey bulls and 4249 Jersey cows. For the evaluation of these national reference populations, GBLUP is currently used to calculate the Australia Genomic Breeding Value on 50 K SNP genotypes. However, even with the current data, prediction accuracy is higher with Bayes R than with GBLUP for some traits and GBLUP is unable to take advantage of the extra information that would be contained in whole-genome sequence data. Therefore, we anticipate moving to a Bayesian method to take advantage of whole-genome sequence data and increase prediction accuracies, and we expect that an EM algorithm will be part of this methodology in order to limit computing time.

In this paper, we used only bulls in the reference and validation sets, to avoid the added complexity of weighting bull and cow trait deviations differently. However, further development of the method described in this paper is needed to include appropriate weighting of phenotypes, multi-breed effects, polygenic effects in the model (as implemented in the MCMC version [19]) and to imbed the Bayesian method within a single-step genetic evaluation [42,43], so that it can be applied to the Australian national dairy evaluations. Also, efficient approaches for inversion of the animal by animal matrix to obtain the PEV need to be investigated to retain the efficiency advantage of emBayesR with very large numbers of animals.

## Conclusions

emBayesR uses an EM-based method to estimate the posterior mode of SNP effects, rather than the MCMC sampling used in BayesR. emBayesR can reduce computing time up to 8-fold compared to BayesR. Results with simulated data and real 630 K SNP dairy cattle data show that genomic prediction accuracy of emBayesR is similar to that of BayesR (0.5% accuracy loss averaged over traits). The computing advantages of emBayesR make it attractive for implementation of genomic prediction in very large datasets.

## Additional files

Additional file 1:
**Calculation of** ${\mathbf{P}}_{\mathbf{ik}}=\mathbf{E}\left({\mathbf{b}}_{\mathbf{ik}}\Big|\mathbf{y},\kern0.5em {\widehat{\mathbf{P}\mathbf{r}}}_{\boldsymbol{k}}\right)$ **.** This file includes the details on how to derive **P**_**ik**_. Additional file 2:
**PEV calculation from GBLUP.** This file includes the details on how to calculate PEV from GBLUP.
