# Supplementary material for: A computationally efficient algorithm for genomic prediction using a Bayesian model
Source: Genet Sel Evol. 2015 Apr 30;47(1):34. doi: 10.1186/s12711-014-0082-4 (PMC4415253; doi:10.1186/s12711-014-0082-4)
Supplement: Additional file 1: — Calculation of ${\mathbf{P}}_{\mathbf{ik}}=\mathbf{E}\left({\mathbf{b}}_{\mathbf{ik}}\Big|\mathbf{y},\kern0.5em {\widehat{\mathbf{P}\mathbf{r}}}_{\boldsymbol{k}}\right)$Pik=Ebik|y,Pr^k . This file includes the details on how to derive P ik. [file 12711_2014_82_MOESM1_ESM.pdf]

Appendix 1 – Calculation of $\mathbf{P_{ik}} = \boldsymbol{E}(\mathbf{b_{ik}}|\mathbf{y}, \widehat{\mathbf{Pr}}_{\boldsymbol{k}})$

In the expectation step of the EM algorithm we require the $E_{b|y}$ of equation 6b. This requires the $\boldsymbol{E}(\mathbf{b_{ik}}|\mathbf{y}, \widehat{\mathbf{Pr}}_{\boldsymbol{k}})$ which is derived in this appendix.

The model is $\mathbf{y} = \mathbf{1}_{\boldsymbol{n}}\mu + \mathbf{Z}_i g_i + \mathbf{u} + \mathbf{e}$,

Then,

$$\boldsymbol{E}_{\mathbf{u}}(b_{ik}|\mathbf{y}, \widehat{\Pr}_k) = p(b_{ik} = 1|\mathbf{y}, \widehat{\Pr}_k)$$

$$= \frac{p(\mathbf{y}|b_{ik}=1) \times p(b_{ik}=1|\widehat{\Pr}_k)}{p(\mathbf{y})}$$

$$\propto p(\mathbf{y}|b_{ik} = 1) \times p(b_{ik} = 1|\widehat{\mathbf{Pr}}_{\boldsymbol{k}}) \quad \text{(A1)}$$

where,

$p(b_{ik} = 1|\widehat{\mathbf{Pr}}_{\boldsymbol{k}}) = \widehat{\mathbf{Pr}}_{\boldsymbol{k}}$ **, and**

$p(\mathbf{y}|b_{ik} = 1) = \frac{1}{\sqrt{|\mathbf{W_k}|}} \exp(-\frac{1}{2}(\mathbf{y} - \mathbf{1}_{\boldsymbol{n}}\mu - \mathbf{u})'\mathbf{W}_k{}^{-1}(\mathbf{y} - \mathbf{1}_{\boldsymbol{n}}\mu - \mathbf{u}))$, so

$log p(\mathbf{y}|b_{ik} = 1) = -0.5\ (log|\mathbf{W}_k| + ((\mathbf{y} - \mathbf{1}_{\boldsymbol{n}}\mu - \mathbf{u})'\mathbf{W}_k{}^{-1}(\mathbf{y} - \mathbf{1}_{\boldsymbol{n}}\mu - \mathbf{u}))$

based on $(y - \mathbf{1}_{\boldsymbol{n}}\mu - \mathbf{u})|(b_{ik} = 1) \sim N(0, \mathbf{W_k})$, and $\mathbf{W_k} = \mathbf{Z_i}\mathbf{Z_i}'\sigma_k^2 + \mathbf{I}\sigma_e^2$.

Therefore,

$$log l_{ik} = log\ p(b_{ik} = 1|\mathbf{y}, \widehat{\Pr}_k) = log p(\mathbf{y}|b_{ik} = 1) + log p(b_{ik} = 1|\widehat{\mathbf{Pr}}_{\boldsymbol{k}}) + constant$$

$constant$ appear on both denominator term and numerator term of equation (A7), and therefore could be ignored.

The expression above for $log p(\mathbf{y}, |b_{ik} = 1)$ involves the unknown $\mathbf{u}$. Therefore, we take the expectation over $\mathbf{u}|\mathbf{y}$. That is,

$log p(\mathbf{y}|b_{ik} = 1) = -0.5\boldsymbol{E}_{\mathbf{u}|\mathbf{y}}\{lo\ g|\mathbf{W}_k| + (\mathbf{y} - \mathbf{1}_{\boldsymbol{n}}\mu - \mathbf{u})'\mathbf{W}_k{}^{-1}(\mathbf{y} - \mathbf{1}_{\boldsymbol{n}}\mu - \mathbf{u})\}$

Only the quadratic form $Q = (\mathbf{y} - \mathbf{1}_{\boldsymbol{n}}\mu - \mathbf{u})'\mathbf{W}_{\mathrm{k}}{}^{-1}(\mathbf{y} - \mathbf{1}_{\boldsymbol{n}}\mu - \mathbf{u})$ of $logp(\mathbf{y}|\mathrm{b}_{\mathrm{ik}} = 1)$ involves $\mathbf{u}$. Therefore, apply Searle's expectation rule[32] for Q as follows:

$$\mathrm{E}_{\hat{\mathbf{u}}}Q = (\mathbf{y} - \mathbf{1}_{\boldsymbol{n}}\mu - \hat{\mathbf{u}})'\mathbf{W}_{\mathrm{k}}{}^{-1}(\mathbf{y} - \mathbf{1}_{\boldsymbol{n}}\mu - \hat{\mathbf{u}}) + \mathrm{tr}(\mathbf{W}_{\mathrm{k}}{}^{-1}PEV((\hat{\mathbf{u}}))$$

Hence,

$$logp(\mathbf{y},|\mathrm{b}_{\mathrm{ik}} = 1) = -0.5\{lo\,g|\mathbf{W}_{\mathrm{k}}| + \mathrm{E}_{\hat{\mathbf{u}}}Q\}$$

$$= -0.5\left\{lo\,g|\mathbf{W}_{\mathrm{k}}| + \mathbf{y}^{\dagger\prime}\mathbf{W}_{\mathrm{k}}{}^{-1}\,\mathbf{y}^{\dagger} + \mathrm{tr}(\mathbf{W}_{\mathrm{k}}{}^{-1}PEV((\hat{\mathbf{u}}))\right\}$$

where, $\mathbf{y}^{\dagger} = (\mathbf{y} - \mathbf{1}_{\boldsymbol{n}}\mu - \hat{\mathbf{u}})$.

Although $\mathbf{W}_{\mathrm{k}}$ is an $n \times n$ matrix. the calculation of $log|\mathbf{W}_{\mathrm{k}}|$ and $\mathbf{W}_{\mathrm{k}}{}^{-1}$ can be simplified by using the Woodbury identity so that

$$\mathbf{W}_{\mathrm{k}}{}^{-1} = \left(\mathbf{Z}_{\mathbf{i}}\mathbf{Z}_{\mathbf{i}}{}'\sigma_{\mathrm{k}}^2 + \mathbf{I}\sigma_{\mathrm{e}}^2\right)^{-1} = \sigma_{\mathrm{e}}^{-2}\left(I - \frac{\mathbf{Z}_{\mathbf{i}}\mathbf{Z}_{\mathbf{i}}'\sigma_{\mathrm{k}}^2}{\sigma_{\mathrm{k}}^2\mathbf{Z}_{\mathbf{i}}'\mathbf{Z}_{\mathbf{i}} + \sigma_{\mathrm{e}}^2}\right) \quad \text{(A2)}$$

$$|\mathbf{W}_{\mathrm{k}}| = \sigma_{\mathrm{e}}^{(2\mathrm{n}-2)}(\sigma_{\mathrm{k}}^2\mathbf{Z}_{\mathbf{i}}'\mathbf{Z}_{\mathbf{i}} + \sigma_{\mathrm{e}}^2), \text{ so}$$

$$log|\mathbf{W}_{\mathrm{k}}| = (2\mathrm{n} - 2)\mathrm{lo\,g}\,\sigma_{\mathrm{e}}^2 + log\left(\sigma_{\mathrm{k}}^2\mathbf{Z}_{\mathbf{i}}'\mathbf{Z}_{\mathbf{i}} + \sigma_{\mathrm{e}}^2\right) \quad \text{(A3)}$$

Such transformation could transfer the inverse calculation of a large matrix $\mathbf{W}_{\mathrm{k}}$ to the multiplication of the vectors, which could reduce the cost for matrix calculation.

Therefore, substitute (A3) and (A4) into $logp(\mathbf{y}|\mathrm{b}_{\mathrm{ik}}, \hat{\mathbf{u}})$ as follow:

$$logp(\mathbf{y}|\mathrm{b}_{\mathrm{ik}} = 1) = -0.5\left\{(\mathrm{n} - 1)\mathrm{lo\,g}\,\sigma_{\mathrm{e}}^2 + \log\left(\sigma_{\mathrm{k}}^2\mathbf{Z}_{\mathbf{i}}'\mathbf{Z}_{\mathbf{i}} + \sigma_{\mathrm{e}}^2\right)\right\}$$

$$-0.5\left\{\left(\mathbf{y}^{*\prime}\mathbf{y}^{*}\right)\sigma_{\mathrm{e}}^{-2} - \left(\mathbf{y}^{*\prime}\mathbf{Z}_{\mathrm{i}}\right)^2\sigma_{\mathrm{k}}^2\sigma_{\mathrm{e}}^{-2}/\left(\sigma_{\mathrm{k}}^2\mathbf{Z}_{\mathbf{i}}'\mathbf{Z}_{\mathbf{i}} + \sigma_{\mathrm{e}}^2\right)\right\}$$

$$-0.5\left\{\mathrm{tr}\left(\mathrm{PEV}(\hat{\mathbf{u}})\right)\sigma_{\mathrm{e}}^{-2} - \mathrm{tr}\left(\mathbf{Z}_{\mathbf{i}}\mathbf{Z}_{\mathbf{i}}'\mathrm{PEV}(\hat{\mathbf{u}})\right)\sigma_{\mathrm{k}}^2\sigma_{\mathrm{e}}^{-2}/\left(\sigma_{\mathrm{k}}^2\mathbf{Z}_{\mathbf{i}}'\mathbf{Z}_{\mathbf{i}} + \sigma_{\mathrm{e}}^2\right)\right\} \quad \text{(A4)}$$

Then,

$$logl_{ik} = logp(\mathbf{y}|\mathrm{b}_{\mathrm{ik}} = 1) + logp(\mathrm{b}_{\mathrm{ik}} = 1|\widehat{\mathbf{Pr}}_{\boldsymbol{k}})$$

$$= -0.5\{2(\mathrm{n} - 1)\mathrm{lo\,g}\,\sigma_{\mathrm{e}}^2 + \log\mathrm{V}\}$$

$$-0.5\left\{(\mathbf{y}^{\dagger\prime}\mathbf{y}^{\dagger})\sigma_e^{-2} - \left(\mathbf{y}^{\dagger\prime}\mathbf{Z_i}\right)^2\sigma_k^2\sigma_e^{-2}/V\right\}$$

$$-0.5\{\mathrm{tr}(\mathrm{PEV}(\hat{\mathbf{u}}))\sigma_e^{-2} - \mathrm{tr}(\mathbf{Z_i}\mathbf{Z_i}'\mathrm{PEV}(\hat{\mathbf{u}}))\sigma_k^2\sigma_e^{-2}/V\}$$

$$+log\mathrm{Pr_k} \tag{A5}$$

where, $\mathbf{y}^{\dagger} = \mathbf{y} - \mathbf{1}_{\boldsymbol{n}}\mu - \hat{\mathbf{u}}$, $V = \sigma_k^2\mathbf{Z_i}'\mathbf{Z_i} + \sigma_e^2$ and n is the number of animals. $\mathrm{PEV}(\hat{\mathbf{u}})$ ($n \times n$ symmetric matrix) could be approximated by $\mathrm{PEV}(\widehat{\mathrm{u}^*})$ as derived in appendix 2 and could be calculated based on GBLUP, outside the iterations of EM algorithm. $\mathrm{tr}(\mathbf{Z_i}\mathbf{Z_i}'\mathrm{PEV}(\hat{\mathbf{u}}))$ means to add up the diagonal elements of symmetric matrix. In other words, we just need to calculate and then add up the diagonal elements of the multiplication of $\mathbf{Z_i}\mathbf{Z_i}'$ (also a $n \times n$ symmetric matrix) and $\mathrm{PEV}(\hat{\mathbf{u}})$. Because $\mathrm{tr}(\mathbf{Z_i}\mathbf{Z_i}'\mathrm{PEV}(\hat{\mathbf{u}}))$ and $\mathrm{tr}(\mathrm{PEV}(\hat{\mathbf{u}}))$ does not change each iterations, they could be calculated once and stored in front of the EM steps.

With the expression for $\mathrm{logl_{ik}} = logp(\mathrm{b_{ik}} = 1|\mathbf{y}, \widehat{\mathbf{Pr}}_{\boldsymbol{k}})$, we can now calculate the probability that each SNP is in one of four normal distributions:

$$P_{ik} = \boldsymbol{E}_{\mathbf{u}}(\mathrm{b_{ik}}|\mathbf{y}, \widehat{\mathrm{Pr}}_k) = \frac{\exp(logl_{ik})}{\sum_{k=1}^{4}\exp(logl_{ik})} \tag{A6}$$
