# Supplementary material for: A computationally efficient algorithm for genomic prediction using a Bayesian model
Source: Genet Sel Evol. 2015 Apr 30;47(1):34. doi: 10.1186/s12711-014-0082-4 (PMC4415253; doi:10.1186/s12711-014-0082-4)
Supplement: Additional file 2: — PEV calculation from GBLUP. This file includes the details on how to calculate PEV from GBLUP. [file 12711_2014_82_MOESM2_ESM.pdf]

Appendix 2 – PEV calculation from GBLUP

In the EM algorithm, we will need the prediction error variance of $\hat{\mathbf{u}}$ (PEV). $\hat{\mathbf{u}}$ is the sum of the estimated effects of all the SNP multiplied by the genotypes for each animal but we approximate its PEV by assuming it is normally distributed and therefore can be calculated by the GBLUP model. That is,

For *n* animals, the phenotype can be modelled by a simplified model:

$$\mathbf{y} = \mathbf{1}_{\boldsymbol{n}}\mu + \mathbf{u}^* + \mathbf{e}$$

Where $\mathbf{e} \sim \mathrm{N}(0, \mathbf{I}\sigma_e^2)$

$\mathbf{u}^*$ is the breeding value for all of the animals($\mathbf{u}^* = \mathbf{Zg}$), $\mathbf{u}^* \sim N(\mathbf{0}, \mathbf{G}\sigma_g^2)$ **,** here **G** is the genomic relationship matrix, $\mathbf{G} = \frac{ZZ'}{2\sum p_i(1-p_i)}$ from [3] **(**p_i is the frequency of the second allele "1" for SNP *i*)**.**

Then, the prediction error variance of $\widehat{\mathbf{u}^*}$ is:

$$\mathrm{PEV}(\widehat{\mathbf{u}^*}) = \mathrm{Var}(\mathbf{u}^* - \widehat{\mathbf{u}^*}) = (\mathbf{G}^{-1}\sigma_g^{-2} + \mathbf{I}\sigma_e^{-2})^{-1} \quad \text{(A7)}$$

In emBayesR, we also use the model

$$\mathbf{y} = \mathbf{1}_{\boldsymbol{n}}\mu + \mathbf{u} + \mathrm{Z_i g_i} + \mathbf{e}$$

where $\mathbf{u} = \mathbf{u}^* - \mathrm{Z_i g_i}$.

but we assume $\mathrm{PEV}(\hat{\mathrm{u}}) = \mathrm{PEV}(\widehat{\mathrm{u}^*})$. In fact u^* differs from u is that u^* includes the effect of the current SNP ($\mathrm{Z_i g_i}$) whereas u does not. Consequently, $\mathrm{PEV}(\hat{\mathrm{u}}) < \mathrm{PEV}(\widehat{\mathrm{u}^*})$. However, the difference should be small because the effect of each SNP is small and the estimated effect is even smaller because it is shrunk especially in the GBLUP model.
